# Rapid and Reliable Conformational Analysis of Glycans by Small Angle X‐Ray Scattering Guided Molecular Dynamics Simulations

**DOI:** 10.1002/cphc.202500323

**Published:** 2025-10-14

**Authors:** Yadiel Vázquez‐Mena, Nishu Yadav, Surusch Djalali, Isabelle Morfin, Martina Delbianco, Yu Ogawa

**Affiliations:** ^1^ CNRS, Cermav University of Grenoble Alpes 38000 Grenoble France; ^2^ Department of Biomolecular Systems Max Planck Institute of Colloids and Interfaces Am Mühlenberg 1 14476 Potsdam Germany; ^3^ Department of Chemistry and Biochemistry Freie Universität Berlin Arnimallee 22 14195 Berlin Germany; ^4^ LiPHY University of Grenoble Alps CNRS 38402 Grenoble France; ^5^ Department of Sustainable and Bioinspired Materials Max Planck Institute of Colloids and Interfaces Am Mühlenberg 1 14476 Potsdam Germany

**Keywords:** conformations, glycans, hairpins, molecular dynamics simulations, small‐angle X‐ray scattering

## Abstract

Glycan conformations play essential roles in biological recognition, immune response, and cellular communication, as well as the properties of carbohydrate‐based materials. Despite their importance, analyzing their secondary structures poses significant challenges due to their inherent molecular flexibility and extensive hydration. Traditional techniques like nuclear magnetic resonance (NMR) and X‐ray crystallography often struggle to capture their dynamic nature accurately. Computational approaches, particularly molecular dynamics (MD) simulations, have emerged as a powerful tool to study glycan conformations, but their accuracy relies heavily on validation against experimental data. In this study, the conformation of glycans in the solution state is investigated by integrating small‐angle X‐ray scattering (SAXS) and MD simulations. By explicitly accounting for the conformational dynamics and hydration effects, the MD simulations accurately predicted the SAXS intensities of two glycan hairpins with similar primary sequences. This approach enables the resolving of their intricate conformational properties, including distinct secondary structures, radii of gyration, and conformational rigidity and dynamics. These findings offer a robust, label‐free analytical strategy for glycan conformational studies, with potential applications in the molecular design of glycan‐based materials and therapeutics.

## Graphical abstract

By explicitly accounting for the conformational dynamics and hydration effects, the molecular dynamics simulations accurately predicted the small‐angle X‐ray scattering intensities of two glycan hairpins with similar primary sequences. This approach enables the resolving their intricate conformational properties, including distinct secondary structures, radii of gyration, and conformational rigidity and dynamics.

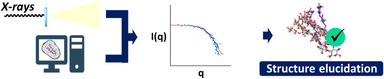

## 1 Introduction

The classification of glycan structures into primary, secondary, and tertiary levels mirrors that of proteins, providing a framework for understanding their structural organization.^[^ ^1^ ^]^ The primary structure refers to the specific sequence of monosaccharide residues linked by glycosidic bonds. The secondary structure describes the spatial arrangements that these sequences can adopt, while the tertiary structure refers to how these arrangements come together to form a compact shape. Glycans can adopt various secondary structures, such as helical^[^ ^2^ ^]^ and ribbon‐like^[^ ^3^ ^]^ conformations, which can further aggregate into more complex higher‐order structures.^[^ ^4^ ^]^ These conformations are important determinants of glycans’ biological properties, including immunomodulatory,^[^ ^5^ ^]^ antitumor,^[^ ^6^ ^]^ antioxidant,^[^ ^7^ ^]^ and anti‐inflammatory.^[^ ^8^, ^9^ ^]^ Thus, glycans with tailored secondary structures have been targeted to tune their biological activity and functionality.^[^ ^10^, ^11^, ^12^ ^]^ Additionally, glycan conformations play a crucial role in the design of 3D architectures, paving the way for glycan‐based materials with tailored physicochemical properties.^[^ ^13^ ^]^

Despite the growing interest in glycan conformation, characterizing the secondary structure of glycans is a challenging task, even for well‐defined synthetic oligosaccharides.^[^ ^14^, ^15^ ^]^ Unlike proteins, which have well‐established secondary structure motifs, glycans exhibit a greater degree of conformational flexibility due to the nature of their glycosidic linkages, weak intramolecular interactions, and hydration effects.^[^ ^13^ ^]^ This conformational complexity makes glycans better described with an ensemble of conformations rather than a single 3D structure. In this context, molecular dynamics (MD) simulations are particularly suited to explore the entire conformational space that a glycan can adopt in solution with atomic resolution.^[^ ^16^, ^17^ ^]^ Still, MD simulations require experimental validation, which often involves employing multiple complementary analytical techniques.

Several methods have been applied to complement MD simulations and study glycan secondary structures, each offering unique insights. Optical rotation measurements help in assessing the overall chiral properties of glycans in solution.^[^ ^18^ ^]^ Circular dichroism (CD) spectroscopy provides information about chiral arrangements in glycans structures and can be used to assess helical or other organized conformations.^[^ ^19^, ^20^ ^]^ However, its application is significantly limited by the absence of chromophores in its molecular structure. Crystallographic analysis is useful for studying crystalline glycan structures, revealing highly ordered molecular arrangements.^[^ ^21^ ^]^ Nevertheless, diffraction‐based methods can only describe the conformation in the crystalline phase, and sample preparation poses a challenge and greatly affects the final results.^[^ ^22^ ^]^

Nuclear magnetic resonance (NMR) spectroscopy is the most commonly used technique to obtain atomic‐level structural details, offering insights into glycosidic bond angles, hydrogen bonding, and overall 3D conformation.^[^ ^23^ ^]^ However, glycan chemical shift degeneracy, particularly in the proton (^1^H) spectrum, often hampers the unambiguous assignment of resonances. To improve spectral resolution and facilitate structural characterization, isotopic labeling (by incorporation of ^13^C and ^15^N enriched monosaccharides)^[^ ^24^ ^]^ and paramagnetic lanthanide complexation^[^ ^25^ ^]^ have been explored. These approaches provide better spectral resolution but require additional synthetic steps and rely on expensive isotopically enriched starting materials.

Electrospray ionization‐scanning tunneling microscopy (ESI‐STM)^[^ ^26^ ^]^ enables the direct imaging of glycan structures, one molecule at a time, offering insights into their surface morphology,^[^ ^27^ ^]^ conformational states,^[^ ^28^ ^]^ and supramolecular organization.^[^ ^29^ ^]^ However, so far, ESI‐STM remains limited to the description of glycan conformations in the gas phase.

Small‐angle scattering (SAS) techniques, including small‐angle X‐ray scattering (SAXS) and small‐angle neutron scattering (SANS),^[^ ^30^ ^]^ can serve as an experimental benchmark to validate MD simulations.^[^ ^31^ ^]^ These methods provide relatively low‐resolution yet essential information about the overall molecular shape, flexibility, and aggregation behavior of glycans in solution. By comparing scattering intensities with those generated from MD simulations, computational models can be either validated or refined to better capture glycan flexibility and hydration effects. Such an approach, so‐called SAS‐guided MD simulation, has been useful in mitigating the risk of overinterpretation during structure refinement of proteins.^[^ ^32^ ^]^

SAXS was employed to analyze a variety of glycans, including κ‐ and ι‐carragenans,^[^ ^33^, ^34^, ^35^, ^36^, ^37^ ^]^ amylose,^[^ ^38^, ^39^ ^]^ gellan gum,^[^ ^40^, ^41^ ^]^ pectic acid,^[^ ^30^ ^]^ carob galactomannan^[^ ^34^ ^]^ and cellulose,^[^ ^42^ ^]^ as well as oligomer samples with degree of polymerization lower than 20, including pullulan,^[^ ^40^ ^]^ alginate (β‐D‐mannopyranuronic acid and α‐L‐gulopyranuronic acid),^[^ ^30^ ^]^ pectic acid,^[^ ^30^ ^]^ and cellulose.^[^ ^43^ ^]^ In most previous studies, glycans with undefined molecular weight were subjected to SAXS measurements. Such polydisperse samples caused blurred scattering profiles due to overlapping contributions from molecules with different lengths, posing challenges to extracting structural details. More recently, SAXS was applied to well‐defined synthetic decasaccharides.^[^ ^44^ ^]^ SAXS measurements combined with atomistic MD simulations allowed describing finer molecular details of these synthetic oligosaccharides, including their conformational flexibility. However, the simulation of scattering intensities relied on a small number of snapshots of static molecular conformations, eliminating the dynamic and statistical information from the comparison. Furthermore, previous studies did not consider the hydration effects in scattering intensity simulations, even though glycans significantly alter the structure and dynamics of surrounding water molecules, thereby affecting the SAXS signals.^[^ ^45^ ^]^

**Figure 1:**
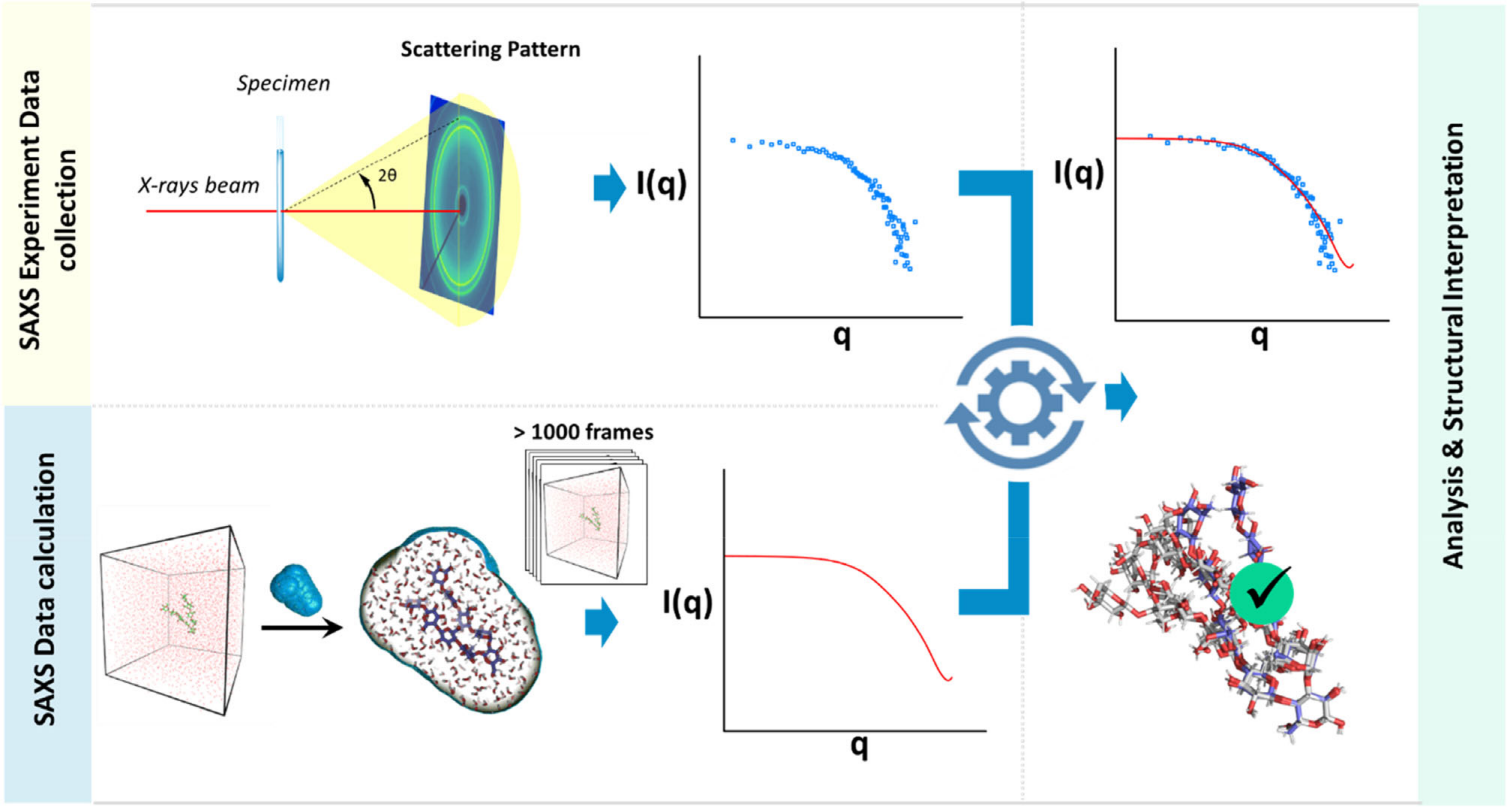
Workflow of SAXS‐MD analysis. A simplified scheme of the acquisition of the scattering pattern to obtain experimental SAXS curves after azimuthal integration and subtraction of the background (top left). A simplified scheme of the construction of the solvation shell and the excluded solvent using a spatial envelope. Such models are finally used to calculate the SAXS curve using GROMACS‐SWAXS considering about 1000 frames related to the same conformation (bottom left). The fitting of the experimental and calculated data to validate the glycan model (right).

In this study, we present a protocol to analyze glycan conformations based on the combination of experimental SAXS measurements and atomistic MD simulations (**Figure** **1**). Unlike previous approaches that relied on static models of glycan molecules, we simulated the SAXS intensity based on the atomistic MD trajectories, taking into account the dynamics of the glycan as well as the contribution from water molecules in the hydration shells. To showcase our protocol, we compared two oligosaccharides with similar primary sequences but different conformational preferences. Our approach quickly discriminates between the two molecules, providing quick validation of computational predictions.

## 2 Results and Discussion

### 2.1 Introduction of the Case Study

As a case study to test our protocol based on the combination of MD and SAXS, we selected two glycan nonasaccharides that only differ in one glycosidic residue. **9mer‐III** has been shown to spontaneously adopt a strand‐loop‐strand secondary structure motif in aqueous solution, analogs to parallel peptide hairpins.^[^ ^46^, ^47^ ^]^ **9mer‐III** conformational stability is encoded in its primary sequence. A trisaccharide analogs of the Le^x^ motif^[^ ^46^, ^47^ ^]^ as turn unit guarantees a rigid conformation stabilized by exo‐anomeric effects, the steric repulsion between the NAc‐group of D‐GlcNAc and L‐Rha, a hydrophobic interaction between the α‐side of L‐Rha and D‐Glc, and a nonconventional CH•••O hydrogen bond between the H‐5 of L‐Rha and O‐5 of Glc.^[^ ^48^ ^]^ This turn unit is connected to two cellulose chains as strands, further stabilizing the folded conformation via strand‐strand interactions.^[^ ^47^ ^]^ **9mer‐II** is an analogue structure in which the L‐Rha unit is replaced by a D‐Glc unit (lacking the nonconventional H‐bond), resulting in higher conformational freedom.^[^ ^46^ ^]^

Both structures were previously analyzed with a combination of NMR spectroscopy techniques.^[^ ^46^, ^47^ ^]^ NMR experiments confirmed the propensity to adopt a closed, compact conformation for **9mer‐III**. However, the severe chemical shift degeneracy and scarcity of interresidue and long‐range nuclear Overhauser effects (NOEs) made this process troublesome. Local structural information (i.e., average distances between nuclei) could only be obtained after the synthesis of ^13^C‐labeled hairpins.^[^ ^46^, ^47^ ^]^ Moreover, NMR analysis of **9mer‐II** only showed a lack of interstrand correlations, likely due to the higher flexibility and less compact geometry. A direct comparison between the two analogs was only possible with diffusion‐ordered spectroscopy (DOSY), allowing us to estimate diffusion behavior and molecular size for the two analogues.

We argue that SAXS may offer a faster, complementary approach to obtain low‐resolution structural information of glycans, bypassing the requirement of ^13^C‐enrichment required for the NMR analysis. SAXS can provide quantitative and qualitative information of the flexibility and overall shape of a solute, as well as important structural information on the dynamic state of a molecule in solution. These results could directly validate all‐atom explicit‐solvent MD simulations, providing a quick readout for glycan secondary structure.

### 2.2 Conformational Analysis using Molecular Dynamics Simulations

**Figure 2:**
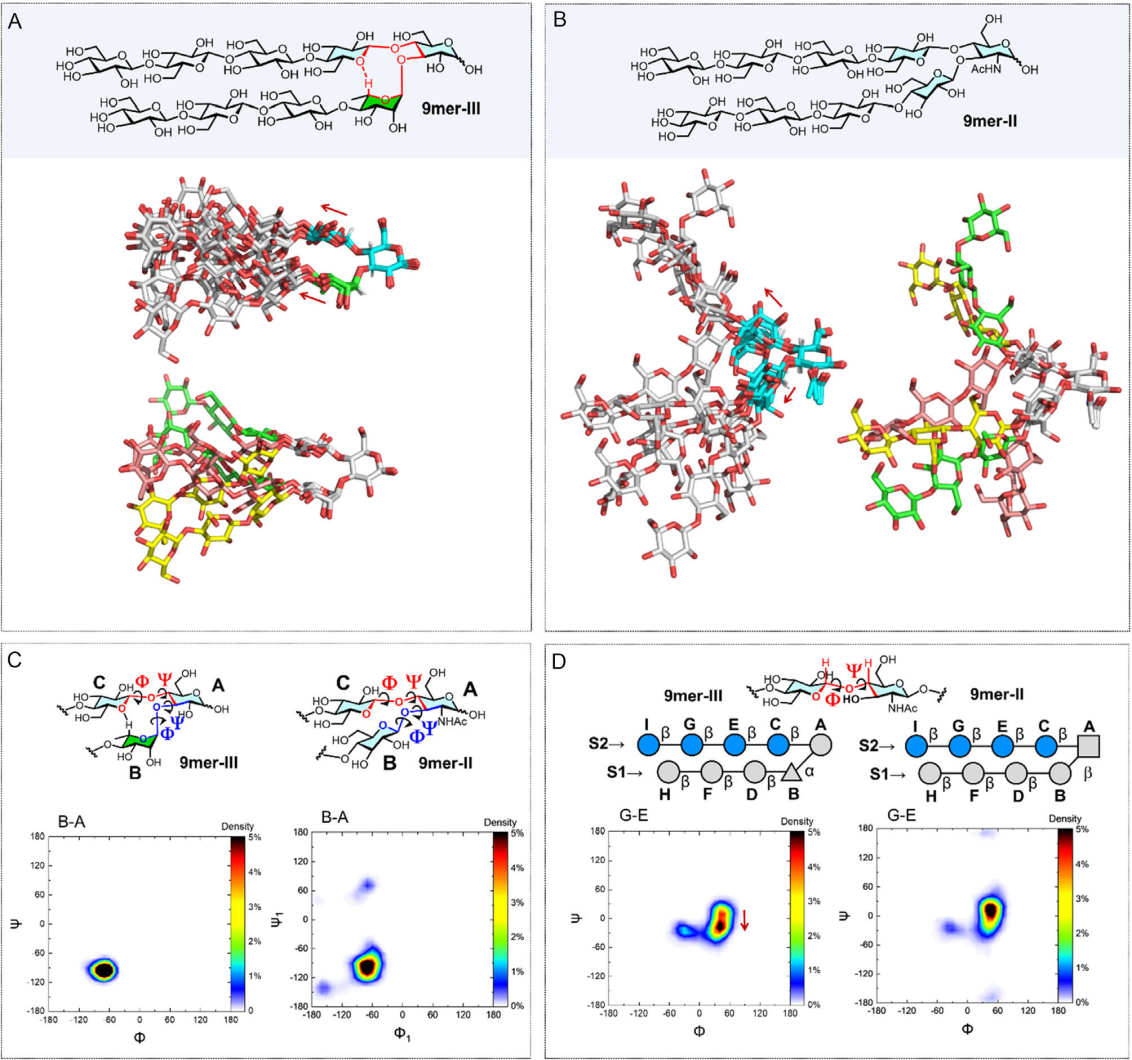
MD simulations analysis of glycan hairpins. A,B) Superpositions of representative snapshots of **9mer‐III** and **9mer‐II** with turn unit highlighted in color (top in A and left in B) and with both strands of each snapshot highlighted in the same color (bottom in A and right in B). Only the hydrogen atoms involved in the glycosidic bonds of the turn unit are shown for simplicity. The red arrows show the direction in which the strands extend according to the geometry adopted by each turn unit. C) Ramachandran plots for the linkage of branched chain at C‐3 in both turn units extracted from the MD simulation show a larger degree of flexibility of **9mer‐II** compared with **9mer‐III**. D) Ramachandran plots for a glycosidic linkage in the strand attach extracted from the MD simulation show the similarity between the global conformation adopted by the cellulose strands in the hairpins, albeit with stronger interactions between strands in **9mer‐III**.

Atomistic MD simulations were carried out to compare the conformational behavior of the two glycans. All structures were simulated for 500 ns based on the GLYCAM06 force field.^[^ ^49^ ^]^ The systems were solvated with the TIP5P water model.^[^ ^50^ ^]^ The MD trajectories showed a clear difference in overall conformation between **9mer‐III** and **9mer‐II**. The latter displayed a larger conformational flexibility and a more extended conformation compared to **9mer‐III**, clearly shown in the representative snapshots (**Figure** **2**A,B).

The Ramachandran plot for the glycosidic linkages (Φ: O^5^’‐C^1^’‐O^n^‐C^n^, Ψ: C^1^’‐O^n^‐C^n^‐C^n−1^) in the turn unit obtained from MD analysis showed that the turn of **9mer‐III** adopts only one conformational state, in contrast with the three main conformational states present for each glycosidic linkage in the turn unit of **9mer‐II** (Figure 2C and 2.5, Supporting Information).^[^ ^46^, ^47^ ^]^ These conformational differences explained the higher conformational rigidity of **9mer‐III** in comparison with **9mer‐II**. The major conformation of the turn unit in **9mer‐III** puts the cellulose strands close in the space, as opposed to the geometry in **9mer‐II**, where the strands are distant, allowing fewer interactions between strands (Figure 2A,B).

The conformation of the strands was compared with that of a single cellulose chain (Figure 1.3, Supporting Information). The torsion angles of the cellulose strands in both glycan hairpins **9mer‐II** and **9mer‐III** showed a main conformational state close to both Φ and Ψ around 0° on the Ramachandran plot, consistent with the results predicted for cellulose oligomers using MD with a different force field (Figure 2D).^[^ ^51^ ^]^ The most‐populated state of the two backbone torsional angles that specify the glycosidic linkage between two D‐Glc monomers (Φ: H^1^’‐C^1^’‐O^4^‐C^4^, Ψ: C^1^’‐O^4^‐C^4^‐H^4^) obtained from this MD analysis was also consistent with experimental data available for crystals of cellulose crystalline allomorphs.^[^ ^51^, ^52^, ^53^ ^]^ At least one intramolecular hydrogen bond for the cellulose strands formed for at least 50% of the simulated time (Figure 2.6, Supporting Information). In general, the atoms involved in such an intramolecular hydrogen bond (Figure 2.6, Supporting Information) are O^3^—H…O^5’^, O^6^—H…O^1’^, O^2’^—H…O^4^, and O^2’^—H…O^6^, similar to those observed in the crystal structures of cellulose.^[^ ^54^ ^]^ These observations supported the extended semirigid ribbon‐like conformation of the strands in both 9mers.

MD simulations predicted a dynamic conformational profile for **9mer‐III,** but with both strands moving synchronously as a result of the observed interstrand interactions (Figure 2A). This observation was supported by the small average distance between opposite residues in each strand from MD simulations.^[^ ^46^, ^47^ ^]^ On the other hand, **9mer‐II** showed higher conformational freedom, with strands moving independently (Figure 2B), higher distances between neighbor residues,^[^ ^46^ ^]^ and practically inexistent interstrand interactions (Figure 2.6, Supporting Information). This behavior is reflected in the Ramachandran plot for the cellulose strands in the two 9mers. While **9mer‐II** matched the plot for the individual cellulose oligomer (Figure 2D), **9mer‐III** shows a maximum conformational population located around (45°, −15°), indicating a shift to negative values for Ψ when compared to isolated cellulose chains in water (45°, 0°) obtained from MD simulations.^[^ ^47^ ^]^ Negative Ψ values indicate a conformation closer to that of the staking cellulose chains in the solid state. For instance, the average value for cellulose I and II in the crystalline state is −22.3° and −27.7°, respectively.^[^ ^53^, ^55^ ^]^

### 2.3 Examination of Scattering Curves

To validate the MD results, we analyzed SAXS intensities of the two samples to detect dynamic macromolecular folding states and conformational changes in solution. As the SAXS pattern is sensitive to the size, shape, and internal electron density distribution of a scattering molecule,^[^ ^56^ ^]^ this analysis will allow us to confirm the differences in conformational structure predicted by MD simulations for these two model oligosaccharides.

**Figure 3:**
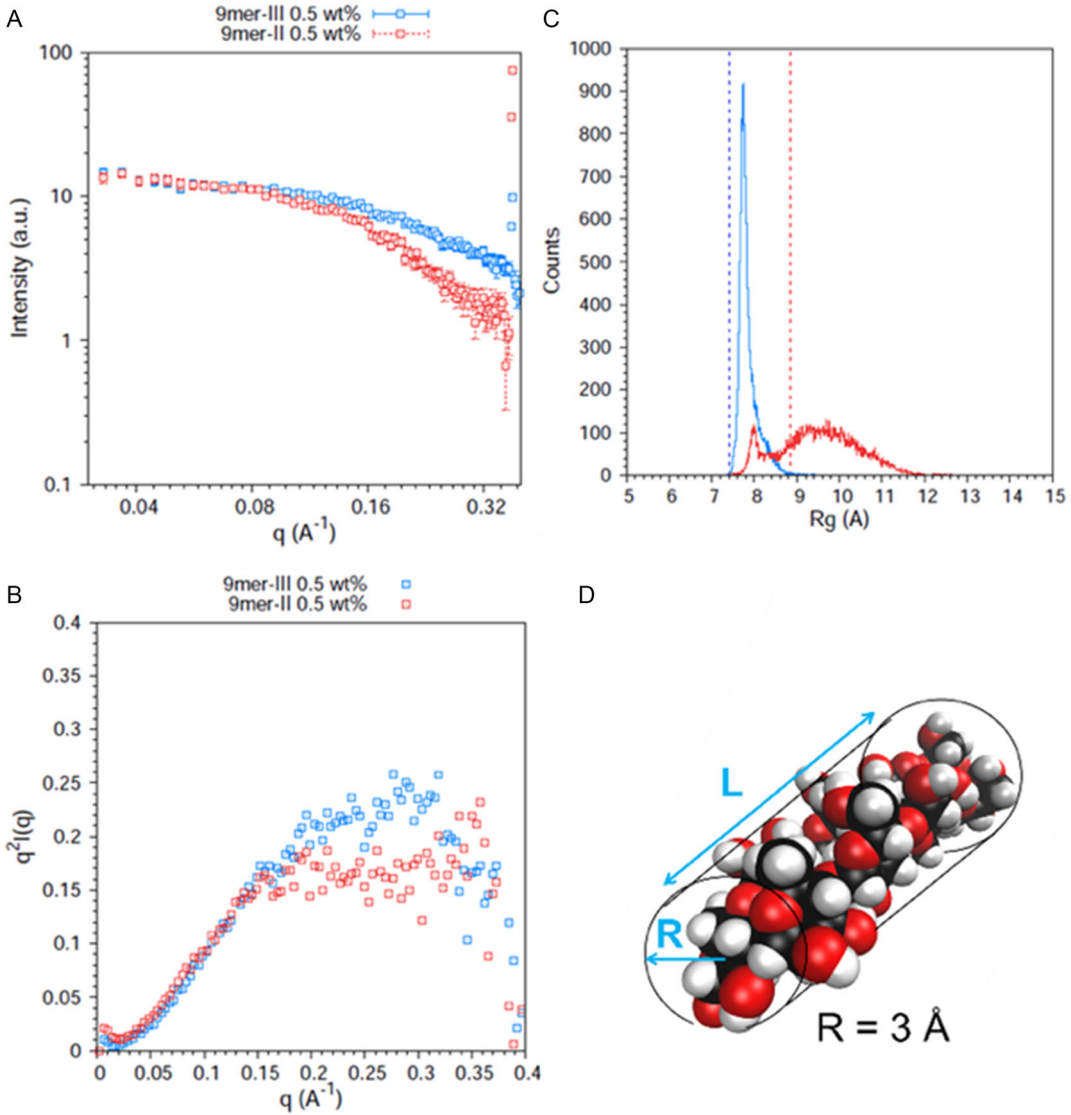
Qualitative SAXS analysis. A) SAXS intensity curves of hairpins **9mer‐III** and **9mer‐II** (5 mg mL^−1^). B) Kratky plots. C) Radius of gyration distribution profiles. Dotted lines denote *R*_g_ values calculated based on Guinier analysis of the experimental SAXS data. D) Model of cylindrical rod representation for **9mer‐III.**

SAXS profiles of the glycan hairpins **9mer‐II** and **9mer‐III** at a concentration of 5 mg/mL are shown in **Figure** **3**A. The SAXS data were relatively noisy due to the low concentration of hairpins in the solutions used to avoid aggregation/precipitation. The SAXS intensity showed a plateau at the low‐*q* region, indicating no aggregation in both glycan samples at this concentration. Such aggregation, characterized by an intensity increase in the lower *q* range, is common in glycan solutions, for example, alginates,^[^ ^57^ ^]^ carrageenan,^[^ ^33^ ^]^ or bacterial polysaccharides.^[^ ^58^ ^]^ The SAXS intensities decreased at a higher *q* range, reflecting the molecular conformations of the hairpins.

Kratky plot allows qualitative evaluation of the flexibility and degree of folding in the glycan hairpins. This analysis has been widely used for the structural characterization of proteins,^[^ ^59^ ^]^ glycans,^[^ ^60^ ^]^ and self‐assembling biomaterials.^[^ ^61^ ^]^ Unfolded, highly flexible macromolecules show a plateau in the Kratky plot at high *q*, while compact, for example, globular proteins are represented by a bell‐shaped peak.

The Kratky plots of **9mer‐II** and **9mer‐III** (Figure 3B) show a broad peak centered at 0.2 Å^−1^, indicating an ordered molecule behavior. This initial analysis clearly indicates that both hairpins **9mer‐II** and **9mer‐III** have certain conformational rigidity and do not adopt disordered or random conformations.

Guinier analysis allowed for estimation of characteristic sizes and shape of the overall conformation of **9mer‐II** and **9mer‐III**. The scattering intensity for highly extended rod‐like objects agrees with the Guinier approximation up to the maximum *q*, with *q*_max_ *R*_g_ being smaller than 1.3. Therefore, the analysis was carried out in the range of *q* ^2^ from 0.008 to 0.06 Å^−2^. The Guinier fit yielded 8.8 Å for **9mer‐III** and 7.9 Å for **9mer‐II** (Figure 1.3, Supporting Information). The radii of gyration estimated by the Guinier approximation agreed with those calculated in the MD simulations of both glycan hairpins (Figure 3C). The radius of gyration of **9mer‐II** is greater than **9mer‐III**, consistent with the hydrodynamic radius obtained from the Stokes–Einstein equation applied to the DOSY results.^[^ ^46^ ^]^ The linearity in the Guinier plot confirmed that both molecules are in the dilute regime with no intermolecular interactions (Figure 1.3, Supporting Information).

Due to the intrinsically rigid backbones and attractive interactions between the strands, hairpins can be assumed to adopt a rigid rod‐like conformation. This model has been successfully applied for the SAXS analysis of cellulose oligomer solutions at high pH.^[^ ^43^ ^]^ Using a cylindrical rod as model (Figure 3D) with a radius *R* = 3 Å, the average length (*L*) of both glycan hairpins is estimated from *R*_g_ with equation, $L^{2}=12R_{g}^{2}–6R^{2}$, yielding *L* = 30 Å and *L* = 26 Å for **9mer‐II** and **9mer‐III**, respectively. Given the length of anhydrous D‐Glc unit (AGU) at 5.15 Å,^[^ ^53^ ^]^ the average length of **9mer‐III** fits a folded conformation with five AGU in length. In contrast, the result obtained for **9mer‐II** does not fit the cylinder model, indicating that **9mer‐II** does not preferentially adopt a closed hairpin conformation with the two cellulose strands in close proximity.

## 3 SAXS Intensity Simulations Based on Explicit‐Solvent Molecular Dynamics Simulations

Simulation of SAXS intensity profiles based on explicit molecular models was necessary to further evaluate differences between the two hairpins. A wide range of methods has been developed for predicting SAXS intensities from atomistic structures. In this work, we used GROMACS‐SWAXS,^[^ ^62^, ^63^ ^]^ an extension of the GROMACS simulation software based on the formalism by Park et al.^[^ ^64^ ^]^ This modification of GROMACS is based on explicit‐solvent MD simulations and has been demonstrated to be a valuable tool for the SAXS analysis of proteins.^[^ ^32^, ^45^, ^62^, ^65^ ^]^ This method computes SAXS profiles using ensembles of structures in MD trajectories, rather than single snapshots, to better represent the dynamic behavior of biomolecules.

**Figure 4:**
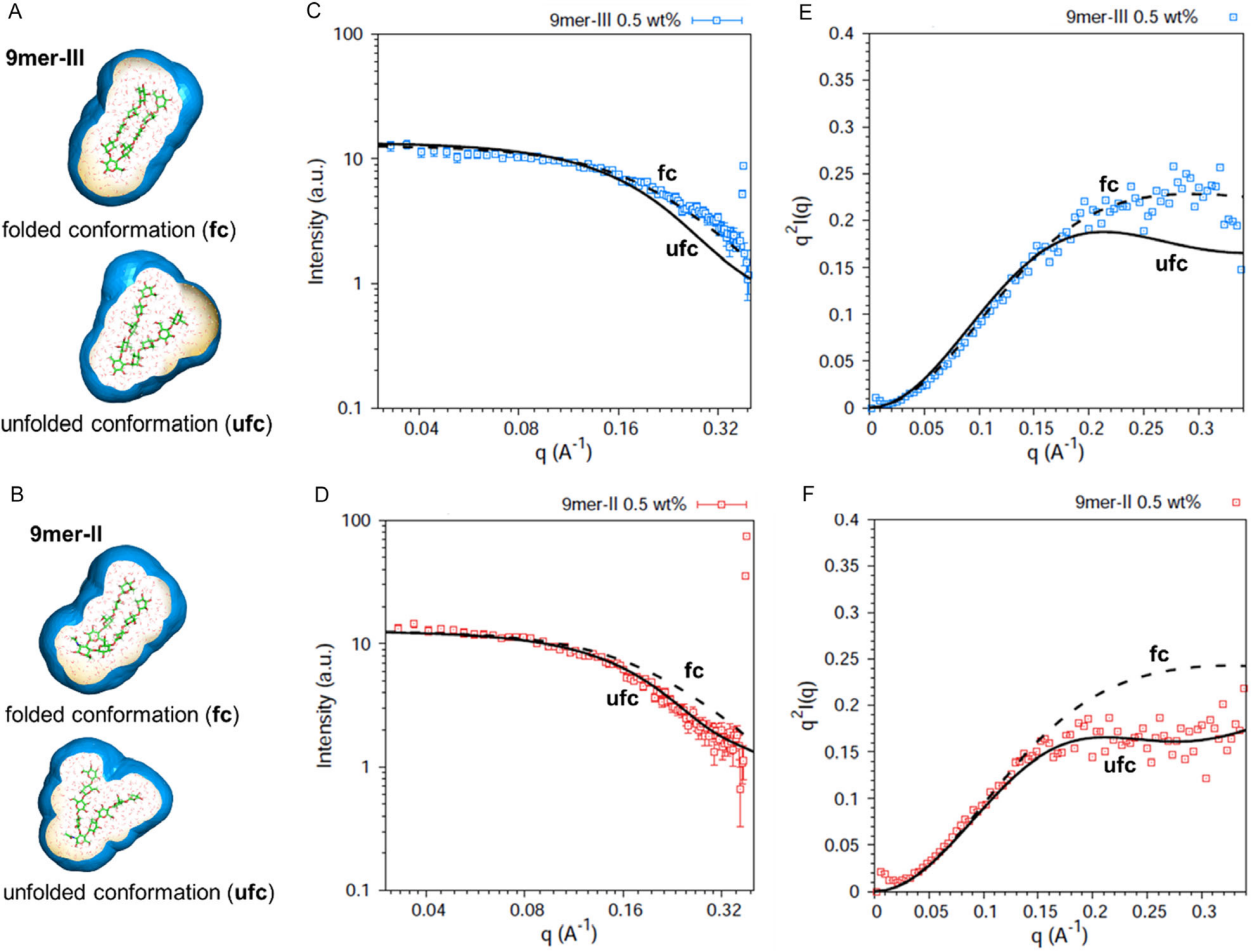
Comparison between calculated and experimental SAXS intensities. A,B) Representations of the two conformational states (folded and unfolded) of hairpins **9mer‐III** and **9mer‐II** obtained from 1–3 ns of MD simulations showing the envelope with water molecules of the solvation layer. C,D) Small‐angle X‐ray scattering intensity. E,F) Kratky plots of hairpins **9mer‐III** and **9mer‐II** (5 mg mL^−1^) and the calculated SAXS intensity of both conformations.

The SAXS profiles were computed from over 1000 consecutive frames taken from selected 1‐3 ns intervals within the 500 ns MD simulations for two conformations of each glycan hairpin, named as the folded (fc) and unfolded (ufc) conformation, respectively (**Figure** **4**A,B and Figure 2.4, Supporting Information). These intervals were chosen after checking the radius of gyration and end‐to‐end distance along the trajectory, which confirmed that the oligomer preferentially adopted different conformations with varying degrees of folding. In the fc conformation, the molecule adopts a compact geometry with the distance between the two terminal C‐4 hydroxyl groups of the two strands within 3–10 Å. On the other hand, the molecule adopts a more extended geometry with interstrand distances between 10–25 Å in the ufc conformation. The solvation envelope was constructed at a distance of 7 Å from the glycan hairpin atoms (for more details, see Section 3.4, Supporting Information).

Figure 4 **C** shows the comparison of experimental and calculated scattering profiles for **9mer‐III**. The experimental SAXS data are in blue, while the calculated intensity is represented with a dashed line for the folded conformation (fc) and a solid line for the unfolded conformation (ufc). The calculated intensity based on the folded conformation agrees well with the experimental data, while those of the unfolded conformation clearly underestimates the intensity values at the *q* range between 0.16 and 0.4 Å^−1^.

An analogous analysis was performed for **9mer‐II** (Figure 4D). The comparison shows that the experimental SAXS data in red fits well with the profile of unfolded conformation (solid line), while the intensity calculated for the folded conformation (dashed line) is higher compared to the experimental data at high *q*.

Calculated scattering profiles based on these two possible conformations also show a clear correspondence with the experimentally determined Kratky plots. While the folded conformation matches the experimental result for **9mer‐III**, the unfolded conformation profile fits that of **9mer‐II** (Figure 4E,F). This analysis unambiguously shows that **9mer‐III** adopts the closed hairpin conformation in solution. In contrast, in **9mer‐II** the absence of a rigid turn unit does not hold the two strands in proximity, promoting an open conformation.

It should be noted that our analysis based on nonbiased MD represents a simplified classification of the glycan conformational space, which is mainly composed of low‐energy states. A more exhaustive characterization would require enhanced sampling approaches, such as well‐tempered metadynamics or multireplica simulations,^[^ ^66^, ^67^ ^]^ to explore the whole free energy landscape. For the present study, however, this simplified description is sufficient to capture the essential features of the glycan hairpin conformation. More complex glycans may benefit from the enhanced sampling strategies.

## 4 Conclusion

In this work, we demonstrated the SAXS‐guided MD simulations as a rapid and efficient method for evaluating the secondary structure of glycans. The method distinguished two oligosaccharides with similar primary sequences but distinct conformational behaviors. Experimental SAXS analyses allowed us to characterize the overall conformation of these two glycan oligomers and validate the MD‐based predictions, unequivocally confirming that **9mer‐III** is more compact than **9mer‐II**. The close agreement between predicted and experimental SAXS profiles highlights the method's accuracy in capturing glycan structures in solution. By explicitly incorporating the conformational dynamics and hydration effects in SAXS intensity predictions, our approach enhances the reliability of the MD‐based conformational study of glycans.

Our method offers an alternative to NMR spectroscopy for glycan conformational analysis by eliminating the need for isotopic labeling. This method's efficiency and simplicity provide a reliable and rapid way to screen glycan conformations in the solution state. Such screening capability may facilitate the molecular designs for glycan‐based materials and drugs.

## Conflict of Interest

The author declares no conflict of interest.

## Supporting information

Supplementary Material

## Data Availability

The data that support the findings of this study are openly available in Edmond at https://doi.org/10.17617/3.2V4C6J.

## References

[1] M. Diener , J. Adamcik , A. Sánchez‐Ferrer , F. Jaedig , L. Schefer , R. Mezzenga , Biomacromolecules 2019, 20, 1731. 30816699 10.1021/acs.biomac.9b00087

[2] M. Tusch , J. Krüger , G. Fels , J. Chem. Theory Comput. 2011, 7, 2919. 26605481 10.1021/ct2005159

[3] A. D. French , S. Pérez , V. Bulone , T. Rosenau , D. Gray , In Encycl. Polym. Sci. Technol. (Ed: H.F. Mark ), Wiley, New York 2018, pp. 1–69.

[4] N. A. Ramsahye , B. J. Howlin , J. Mol. Model. 2000, 6, 477.

[5] A. Varki , Glycobiology 2017, 27, 3. 27558841 10.1093/glycob/cww086 PMC5884436

[6] L. Chen , G. Huang , Curr. Drug Targets 2018, 19, 89. 28676001 10.2174/1389450118666170704143018

[7] J. Wang , S. Hu , S. Nie , Q. Yu , M. Xie , Oxid. Med. Cell. Longev. 2016, 2016, 5692852. 26682009 10.1155/2016/5692852 PMC4670676

[8] B. Mallik , D. Morikis , Curr. Proteomics 2006, 3, 259.

[9] Carbohydrates As Drugs (Eds: P. H. Seeberger , C. Rademacher ), Springer International Publishing, Cham 2014.

[10] S. M. Muthana , C. T. Campbell , J. C. Gildersleeve , ACS Chem. Biol. 2012, 7, 31. 22195988 10.1021/cb2004466 PMC3262866

[11] A. Axer , R. P. Jumde , S. Adam , A. Faust , M. Schäfers , M. Fobker , J. Koehnke , A. K. H. Hirsch , R. Gilmour , Chem. Sci. 2021, 12, 1286. 10.1039/d0sc04297h PMC8179167 34163891

[12] R. Hevey , Chem. – Eur. J. 2021, 27, 2240. 32901973 10.1002/chem.202003135

[13] S. Djalali , N. Yadav , M. Delbianco , Nat. Rev. Mater. 2024, 9, 190.

[14] R. J. Woods , Chem. Rev. 2018, 118, 8005. 30091597 10.1021/acs.chemrev.8b00032 PMC6659753

[15] Y. Yu , M. Delbianco , Chem. – Eur. J. 2020, 26, 9814. 32329095 10.1002/chem.202001370 PMC7496230

[16] S. Perez , O. Makshakova , Chem. Rev. 2022, 122, 15914. 35786859 10.1021/acs.chemrev.2c00060

[17] E. Fadda , Curr. Opin. Chem. Biol. 2022, 69, 102175. 35728307 10.1016/j.cbpa.2022.102175

[18] B. P. Westberry , M. Rio , M. R. Waterland , M. A. K. Williams , Carbohydr. Polym. 2024, 333, 121975. 38494229 10.1016/j.carbpol.2024.121975

[19] W. C. Johnson , In Adv. Carbohydr. Chem. Biochem., Elsevier, New York 1987, pp. 73–124. 3324668

[20] F. E. Stanley , A. M. Stalcup , Anal. Bioanal. Chem. 2011, 399, 701. 20953771 10.1007/s00216-010-4272-9

[21] Polysaccharides: Structural Diversity and Functional Versatility, *Second Ed.* (Ed: S. Dumitriu ), CRC Press, Boca Raton, FL 2004.

[22] Y. Ogawa , J.‐L. Putaux , Y. Nishiyama , Curr. Opin. Chem. Biol. 2022, 70, 102183. 35803025 10.1016/j.cbpa.2022.102183

[23] H.‐Y.‐Y. Yao , J.‐Q. Wang , J.‐Y. Yin , S.‐P. Nie , M.‐Y. Xie , Food Res. Int. 2021, 143, 110290. 33992390 10.1016/j.foodres.2021.110290

[24] M. Delbianco , A. Kononov , A. Poveda , Y. Yu , T. Diercks , J. Jiménez‐Barbero , P. H. Seeberger , J. Am. Chem. Soc. 2018, 140, 5421. 29624385 10.1021/jacs.8b00254

[25] A. Canales , A. Mallagaray , J. Pérez‐Castells , I. Boos , C. Unverzagt , S. André , H. Gabius , F. J. Cañada , J. Jiménez‐Barbero , Angew. Chem. Int. Ed. 2013, 52, 13789. 10.1002/anie.201307845 24346952

[26] S. Rauschenbach , M. Grabarics , M. Delbianco , J. Cortes , C. Schön , N. Tarrat , X. Wu , K. Anggara , In Glycoprotein Anal. (Ed.: W.B. Struwe ), Royal Society Of Chemistry, London 2024, pp. 329.

[27] K. Anggara , Y. Zhu , M. Delbianco , S. Rauschenbach , S. Abb , P. H. Seeberger , K. Kern , J. Am. Chem. Soc. 2020, 142, 21420. 33167615 10.1021/jacs.0c09933 PMC7760097

[28] X. Wu , M. Delbianco , K. Anggara , T. Michnowicz , A. Pardo‐Vargas , P. Bharate , S. Sen , M. Pristl , S. Rauschenbach , U. Schlickum , S. Abb , P. H. Seeberger , K. Kern , Nature 2020, 582, 375. 32555487 10.1038/s41586-020-2362-1

[29] J. Seibel , G. Fittolani , H. Mirhosseini , X. Wu , S. Rauschenbach , K. Anggara , P. H. Seeberger , M. Delbianco , T. D. Kühne , U. Schlickum , K. Kern , Angew. Chem. 2023, 135, e202305733. 10.1002/anie.202305733 37522820

[30] B. W. Mansel , T. M. Ryan , H.‐L. Chen , L. Lundin , M. A. K. Williams , Chem. Phys. Lett. 2020, 739, 136951.

[31] R. P. Rambo , J. A. Tainer , Biophys. J. 2015, 108, 2421. 25992719 10.1016/j.bpj.2015.04.023 PMC4457040

[32] L. Chatzimagas , J. S. Hub , Methods Enzymol., Elsevier, New York 2023, pp. 23–54. 10.1016/bs.mie.2022.09.014 36641209

[33] B. Denef , N. Mischenko , M. H. Koch , H. Reynaers , Int. J. Biol. Macromol. 1996, 18, 151. 8729026 10.1016/0141-8130(95)01056-4

[34] T. Turquois , C. Rochas , F. Taravel , J. L. Doublier , M. Axelos , Biopolymers 1995, 36, 559.

[35] B. P. Westberry , B. W. Mansel , T. M. Ryan , L. Lundin , M. A. K. Williams , Carbohydr. Polym. 2022, 296, 119958. 36088000 10.1016/j.carbpol.2022.119958

[36] P. Hernandez‐Cerdan , B. W. Mansel , A. Leis , L. Lundin , M. A. K. Williams , Biomacromolecules 2018, 19, 989. 29381344 10.1021/acs.biomac.7b01773

[37] Y. Yuguchi , T. T. Thu Thuy , H. Urakawa , K. Kajiwara , Food Hydrocoll. 2002, 16, 515.

[38] D. Braga , E. Ferracini , A. Ferrero , A. Ripamonti , D. A. Brant , G. S. Buliga , A. Cesàro , Int. J. Biol. Macromol. 1985, 7, 161.

[39] P. Roblin , G. Potocki‐Véronèse , D. Guieysse , F. Guerin , M. A. V. Axelos , J. Perez , A. Buleon , Biomacromolecules 2013, 14, 232. 23198782 10.1021/bm301651y

[40] I. Dogsa , J. Štrancar , P. Laggner , D. Stopar , Polymer 2008, 49, 1398.

[41] J. H.‐Y. Liu , D. A. Brant , S. Kitamura , K. Kajiwara , M. Mimura , Macromolecules 1999, 32, 8611.

[42] J. Hagman , L. Gentile , C. M. Jessen , M. Behrens , K.‐E. Bergqvist , U. Olsson , Cellulose 2017, 24, 2003.

[43] F. Jiang , X. Zhang , W. Hwang , Y. Nishiyama , R. M. Briber , H. Wang , Appl. Surf. Sci. 2021, 537, 147783.

[44] S. Jo , D. Myatt , Y. Qi , J. Doutch , L. A. Clifton , W. Im , G. Widmalm , J. Chem. Phys. Chem. 2018, 122, 1169. 10.1021/acs.jpcb.7b11085 29268602

[45] C. J. Knight , J. S. Hub , Nucleic Acids Res. 2015, 43, W225. 25855813 10.1093/nar/gkv309 PMC4489308

[46] G. Fittolani , T. Tyrikos‐Ergas , A. Poveda , Y. Yu , N. Yadav , P. H. Seeberger , J. Jiménez‐Barbero , M. Delbianco , Nat. Chem. 2023, 15, 1461. 37400598 10.1038/s41557-023-01255-5 PMC10533408

[47] N. Yadav , S. Djalali , A. Poveda , M. G. Ricardo , P. H. Seeberger , J. Jiménez‐Barbero , M. Delbianco , J. Am. Chem. Soc. 2024, 146, 6369. 38377472 10.1021/jacs.4c00423 PMC10921397

[48] T. Aeschbacher , M. Zierke , M. Smieško , M. Collot , J. Mallet , B. Ernst , F. H.‐T. Allain , M. Schubert , Chem. – Eur. J. 2017, 23, 11598. 28654715 10.1002/chem.201701866

[49] K. N. Kirschner , A. B. Yongye , S. M. Tschampel , J. González‐Outeiriño , C. R. Daniels , B. L. Foley , R. J. Woods , J. Comput. Chem. 2008, 29, 622. 17849372 10.1002/jcc.20820 PMC4423547

[50] M. W. Mahoney , W. L. Jorgensen , J. Chem. Phys. 2000, 112, 8910.

[51] C. S. Pereira , D. Kony , R. Baron , M. Müller , W. F. Van Gunsteren , P. H. Hünenberger , Biophys. J. 2006, 90, 4337. 16581848 10.1529/biophysj.106.081539 PMC1471844

[52] A. D. French , D. W. Montgomery , N. T. Prevost , J. V. Edwards , R. J. Woods , Carbohydr. Polym. 2021, 264, 118004. 33910736 10.1016/j.carbpol.2021.118004 PMC8607818

[53] Y. Nishiyama , P. Langan , H. Chanzy , J. Am. Chem. Soc. 2002, 124, 9074. 12149011 10.1021/ja0257319

[54] T. Shen , P. Langan , A. D. French , G. P. Johnson , S. Gnanakaran , J. Am. Chem. Soc. 2009, 131, 14786. 19824731 10.1021/ja9034158

[55] P. Langan , Y. Nishiyama , H. Chanzy , J. Am. Chem. Soc. 1999, 121, 9940.

[56] A. G. Kikhney , D. Svergun , FEBS Lett. 2015, 589, 2570. 26320411 10.1016/j.febslet.2015.08.027

[57] B. T. Stokke , K. I. Draget , O. Smidsrød , Y. Yuguchi , H. Urakawa , K. Kajiwara , Macromolecules 2000, 33, 1853.

[58] S. Khan , J. Birch , P. Harris , M.‐R. Van Calsteren , R. Ipsen , G. H. J. Peters , B. Svensson , K. Almdal , Biomacromolecules 2017, 18, 747. 28042938 10.1021/acs.biomac.6b01597

[59] P. Bernadó , D. I. Svergun , Mol BioSyst 2012, 8, 151. 21947276 10.1039/c1mb05275f

[60] K. Kajiwara , I. Wataoka , In Polysacch. Hydrogels Charact. Biomed. Appl., Jenny Stanford Publishing, Singapore 2015, pp. 265–323.

[61] R. Balu , J. Whittaker , J. P. Mata , N. K. Dutta , N. R. Choudhury , In ACS Symp. Ser. (Eds: F. Horkay , J.F. Douglas , E. Del Gado ), American Chemical Society, Washington, DC 2018, pp. 71–89.

[62] P. Chen , J. S. Hub , Biophys. J. 2014, 107, 435. 25028885 10.1016/j.bpj.2014.06.006 PMC4104061

[63] J. Hub , “GROMACS‐SWAXS”, can be found under https://biophys.uni‐saarland.de/software/gromacs‐swaxs/, 2023 (accessed: 2025).

[64] S. Park , J. P. Bardhan , B. Roux , L. Makowski , J. Chem. Phys. 2009, 130, 134114. 19355724 10.1063/1.3099611 PMC2852435

[65] L. Chatzimagas , J. S. Hub , ArXiv Prepr. ArXiv220404961 2022.

[66] A. Barducci , G. Bussi , M. Parrinello , Phys. Rev. Lett. 2008, 100, 020603. 18232845 10.1103/PhysRevLett.100.020603

[67] L. Wang , R. A. Friesner , B. J. Berne , J. Phys. Chem. B 2011, 115, 9431. 21714551 10.1021/jp204407d PMC3172817

