# Supplementary material for: Rapid and Reliable Conformational Analysis of Glycans by Small Angle X‐Ray Scattering Guided Molecular Dynamics Simulations
Source: Chemphyschem. 2025 Oct 14;26(23):e202500323. doi: 10.1002/cphc.202500323 (PMC12677716; doi:10.1002/cphc.202500323)
Supplement: Supplementary file 1 — Supplementary Material [file CPHC-26-e202500323-s001.pdf]

Conformational Analysis of Glycan Hairpins Using Small-angle X-rays Scattering and Molecular Dynamic Simulation

Table of contents

1 Synthesis and SAXS analysis of 9mer-III and 9mer-II 2
1.1 General materials and methods 2
1.2 Guinier plot of 9mer-III and 9mer-II with different concentration 3
1.3 Guinier plot of 9mer-III and 9mer-II (5 mg/mL) 4
1.4 SAXS curve and Kratky plot of 9mer-III and 9mer-II (5 mg/mL) 5
2 Molecular dynamics simulation 6
2.1 General materials and methods 6
2.2 RMSD, End-to-End Distance and Radius of Gyration of 9mer-III and 9mer-II. 7
2.3 Radial pair distribution functions of 9mer-III and 9mer-II 8
2.5 Ramachandran plots[2] 11
2.6 Intra-strands and inter-strands hydrogen bond pairs extracted from the MD simulation 13
2.7 Comparison of RMSD, End-to-End Distance and Radius of Gyration profiles of 9mer-II for 3 replicates 17
2.8 Comparison of RMSD, End-to-End Distance and Radius of Gyration profiles of 9mer-III for 3 replicates 18
2.9 Comparison of Inter-residue Distance of 9mer-II between center of mass of homologous residues in both strands 20
2.10 Comparison of Inter-residue Distance of 9mer-III between center of mass of homologous residues in both strands 21
3 SAXS calculation 22
3.1 General materials and methods 22
3.2 Calculated SAXS intensity curves of two representative conformations of 9mer-II and 9mer-III using different envelopes 23
3.3 Radius of gyration and intensity at q=0 vs. solvation layer. 29
3.4 Analysis of the solvation layer 29

1 Synthesis and SAXS analysis of 9mer-III and 9mer-II

1.1 General materials and methods

Both hairpins were synthesized by AGA on solid support using previously reported conditions.[1,2]

X-ray scattering experiments were performed at the D2AM beamline of the European Synchrotron Radiation Facility (ESRF). Samples of **9mer-II** and **9mer-III** at concentrations 0.5, 0.1, 0.05 and 0.01% wt at 25 ^{o}C were sealed in glass capillaries and mounted on a motorized sample changer. They were exposed to monochromatic X-rays of 15.8 eV (λ = 0.7847 Å). The scattering intensity was measured using two-dimensional pixel detectors (XPAD-WOS and D5). The data processing was performed using pyFAI software. Intensity and Rg analysis were done using Gnuplot software.

1.2 Guinier plot of 9mer-III and 9mer-II with different concentration

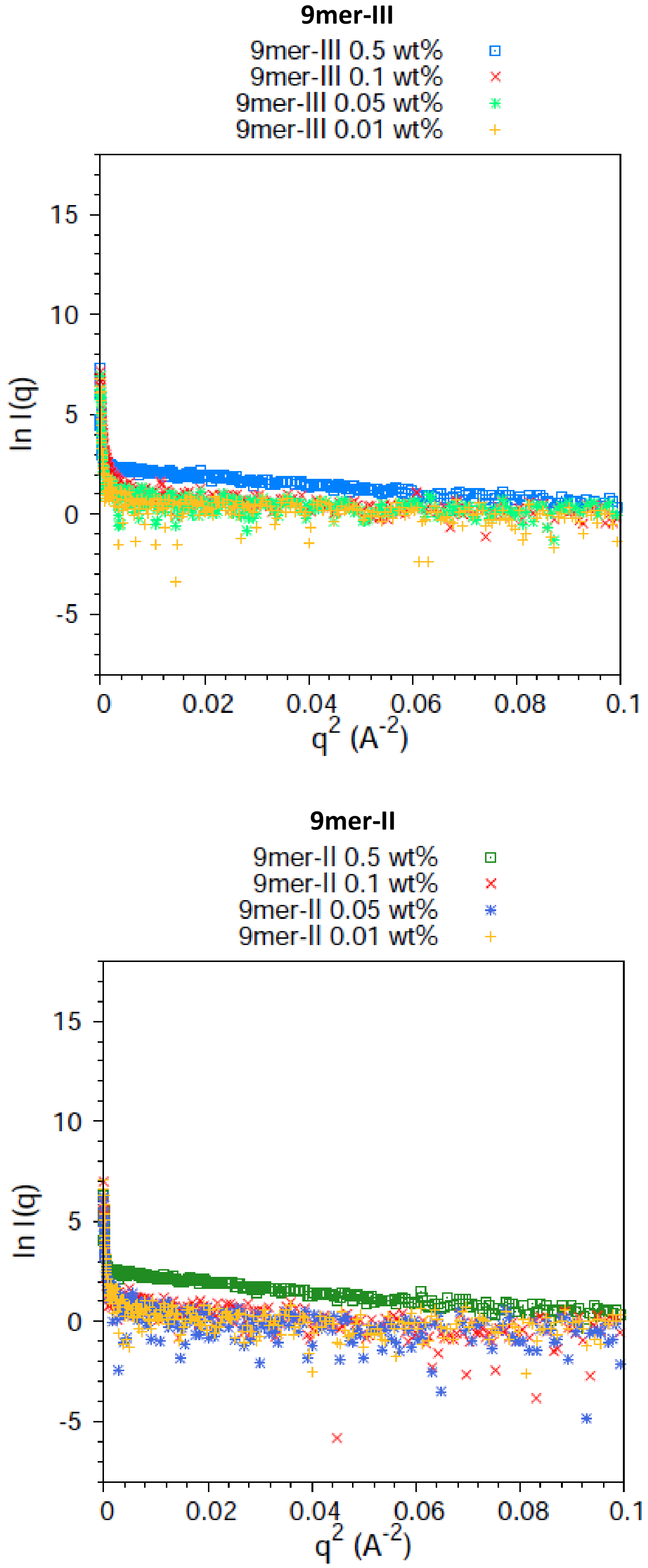

Figure 1 Guinier plot of **9mer-III** and **9mer-II** with different concentration.

1.3 Guinier plot of 9mer-III and 9mer-II (5 mg/mL)

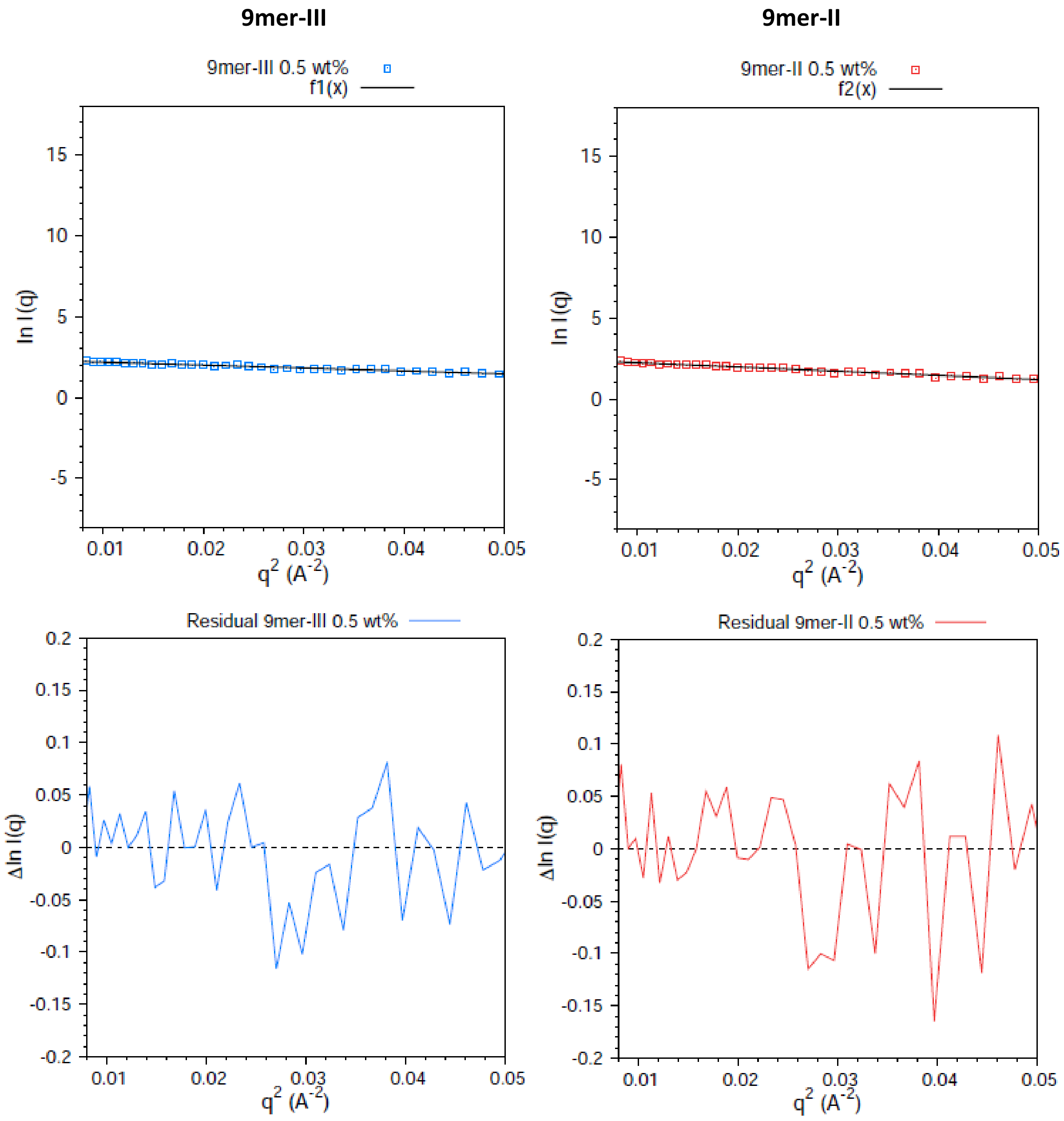

Figure 2 Guinier plot of **9mer-III** and **9mer-II** (5 mg/mL) and residual of fit.

The absence of negative effects in the analysis, such as aggregation and interparticle repulsion, was confirmed by the residual of fit. The residual of both samples shows flat and randomly distributed about zero.

Table of curve fitting of natural logarithm of I(q) vs. q^2 plots of Guinier law-SAXS measurements of glycan hairpins 9mer-II and 9mer-III.

	9mer-III		9mer-II	
	**Value**	**Error**	**Value**	**Error**
**m**	-18.34	0.45	-26.08	0.66
**n**	2.36	0.01	2.49	0.02
**R_g (Å)**	7.42	0.08	8.84	0.07
**RMSE**	0.043		0.062	

1.4 SAXS curve and Kratky plot of 9mer-III and 9mer-II (5 mg/mL)

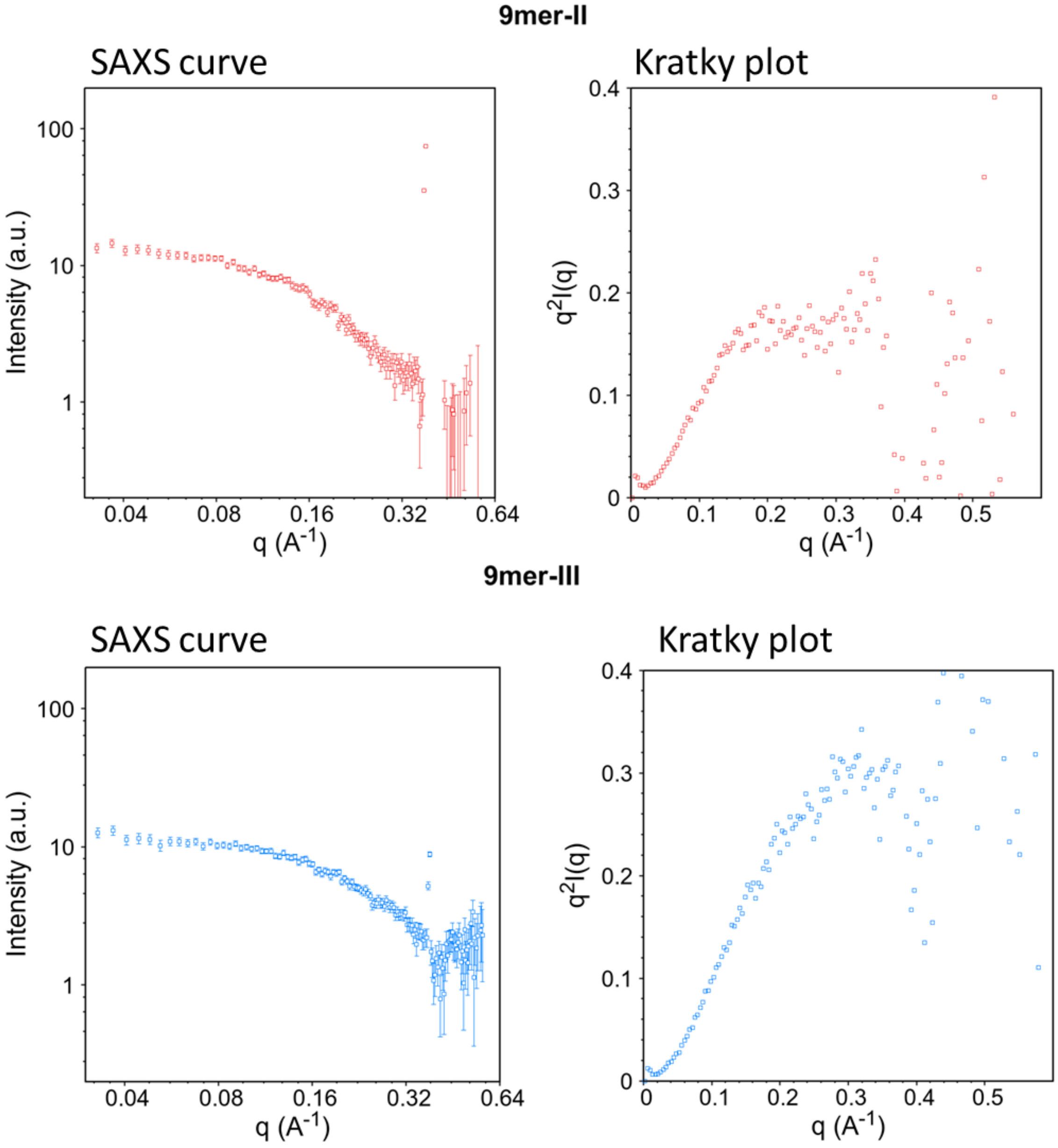

Figure 3 SAXS curve and Kratky plot of **9mer-III** and **9mer-II** (5 mg/mL).

2 Molecular dynamics simulation

2.1 General materials and methods

All-atom MD simulations were performed using GROMACS ver.2022[3]. The oligosaccharides were modelled using a modified version of GLYCAM06[4,5] and the system was solvated with TIP5P[6] water molecules. The topology was converted to GROMACS format using the glycam2gmx.pl script and solvated using GROMACS tools. The systems were kept at a constant temperature of 300 K using a Nosé-Hoover thermostat[7,8] and at constant pressure of 1 bar with the Parrinello-Rahman Barostat[9,10]. Non-bonded interactions were cut off at 1.4 nm, and long-range electrostatics were calculated using the particle mesh Ewald method[11]. Bonds involving hydrogens were constrained using the LINCS[12] algorithm to allow a 2 fs timestep for the solution of the equation of motion. Water molecules were kept rigid with SETTLE[13] algorithm. After energy minimization (steepest descent algorithm) and before the production run, the systems were equilibrated at 300 K for 50 ns in a canonical (NVT) ensemble (constant number of particles N, volume V and temperature T) and subsequently at 300 K and 1 atm for 50 ns in an isothermal-isobaric (NPT) ensemble (constant number of particles N, pressure P and temperature T). All the modelled structures were simulated on three independent replicates of 500 ns each (1.5 μs in total), starting from different velocities. Short 1-3 ns MD intervals were selected as representative models for the SAXS analysis after checking the radius of gyration and end-to-end distance along the trajectory of 500 ns MD simulations.

2.2 RMSD, End-to-End Distance and Radius of Gyration of 9mer-III and 9mer-II.

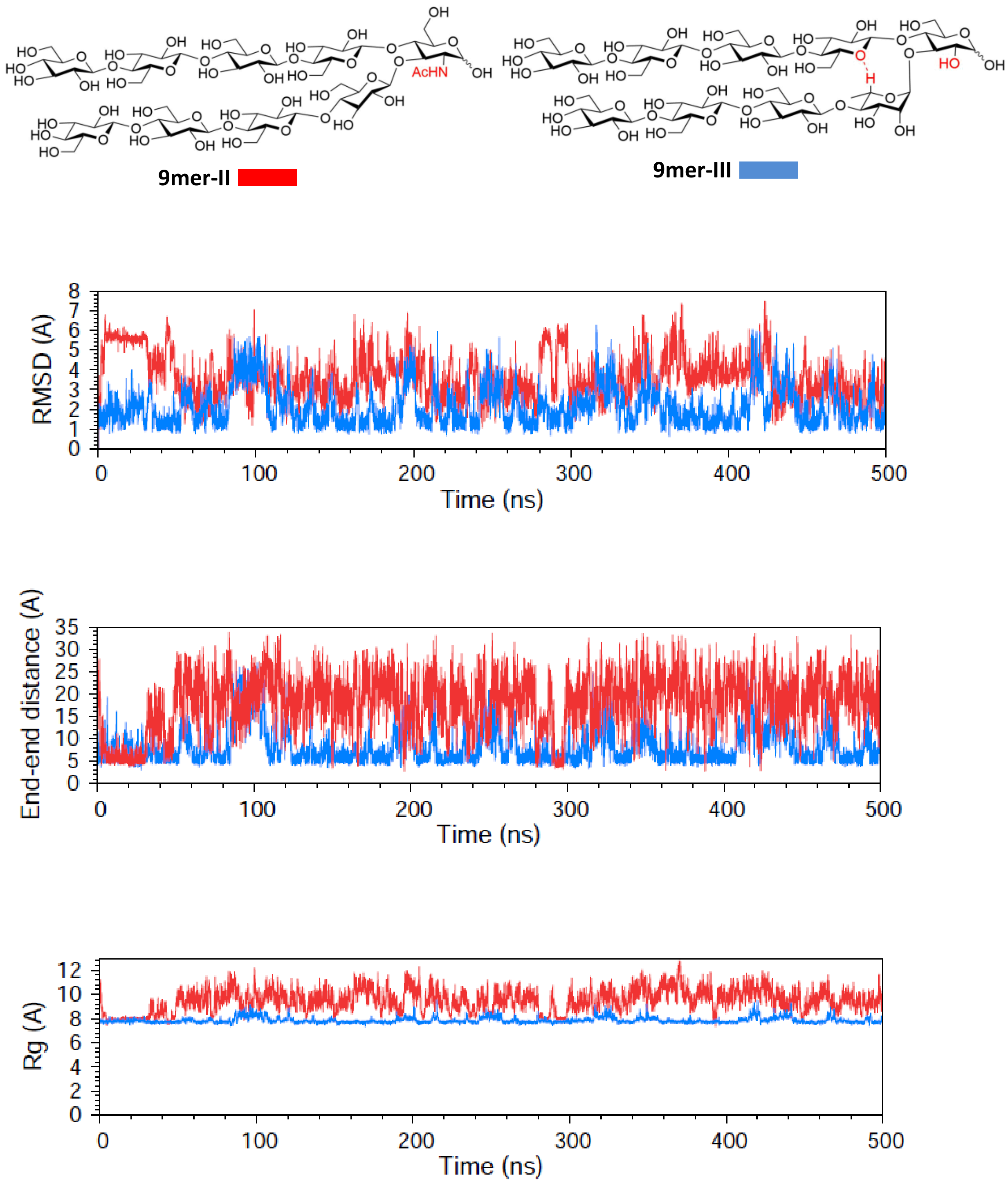

Figure 4 RMSD, End-to-End Distance and Radius of Gyration of **9mer-III** and **9mer-II**. The End-to-End distance was calculated using the C-4 hydroxy group of the non-reducing ends in both oligosaccharides.

2.3 Radial pair distribution functions of 9mer-III and 9mer-II

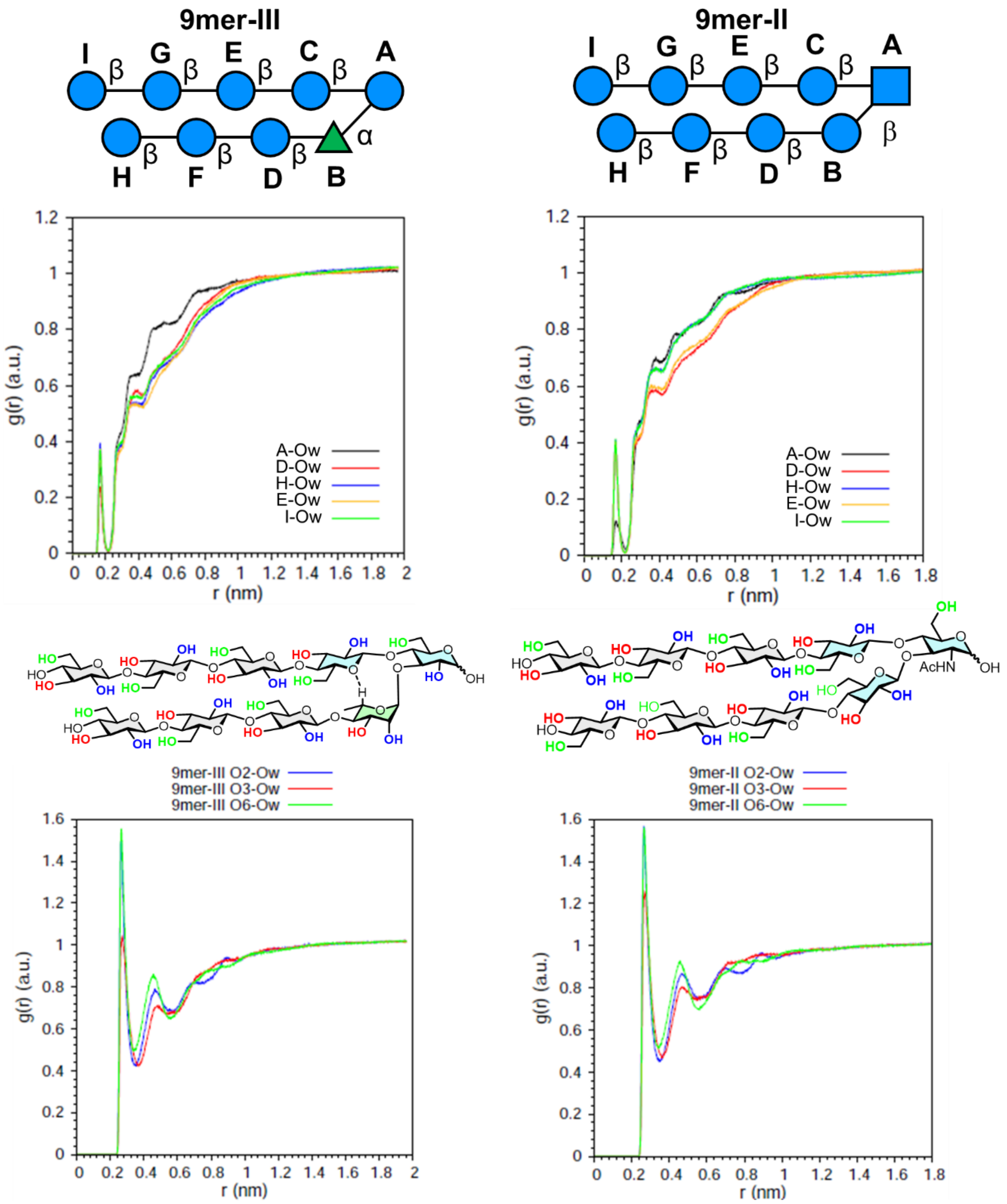

Figure 5 Radial pair distribution functions of **9mer-III** and **9mer-II**. The top graphs show g(r) calculated from the center of mass of selected residues (A, D, H, E and I), while the bottom graphs show g(r) calculated from C-3, C-4 and C-6 hydroxyl groups across oligosaccharide residues, highlighted in different colors.

2.4 End-to-End Distance of selected intervals of 9mer-III and 9mer-II MD simulations

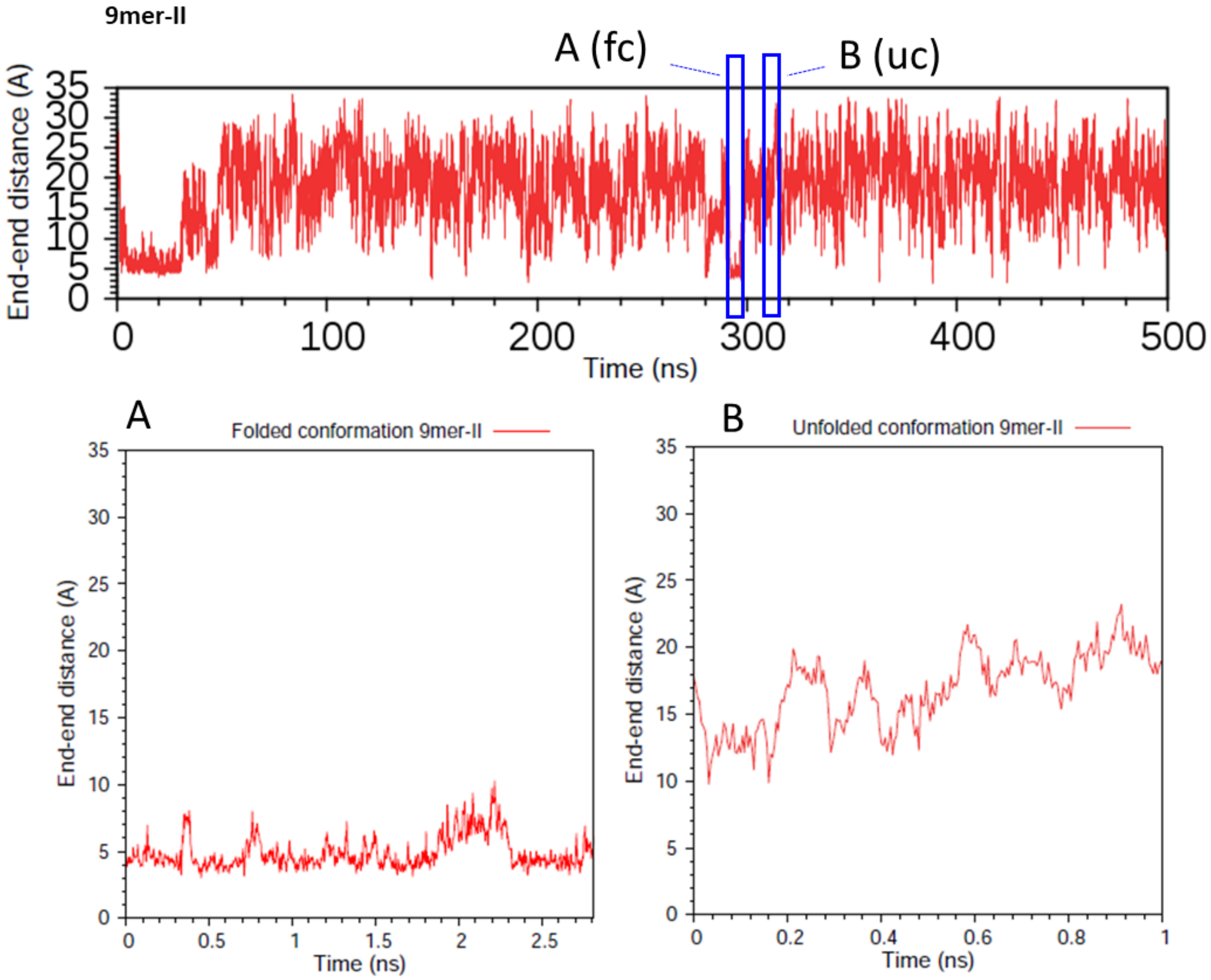

Figure 6 End-to-End Distance of selected intervals of **9mer-II** MD simulations. The top panels indicate the time ranges from the full trajectory used to define interval A (folded conformation model) and interval B (unfolded conformation model). The End-to-End distance was calculated using the C-4 hydroxy group of the non-reducing ends in both oligosaccharides.

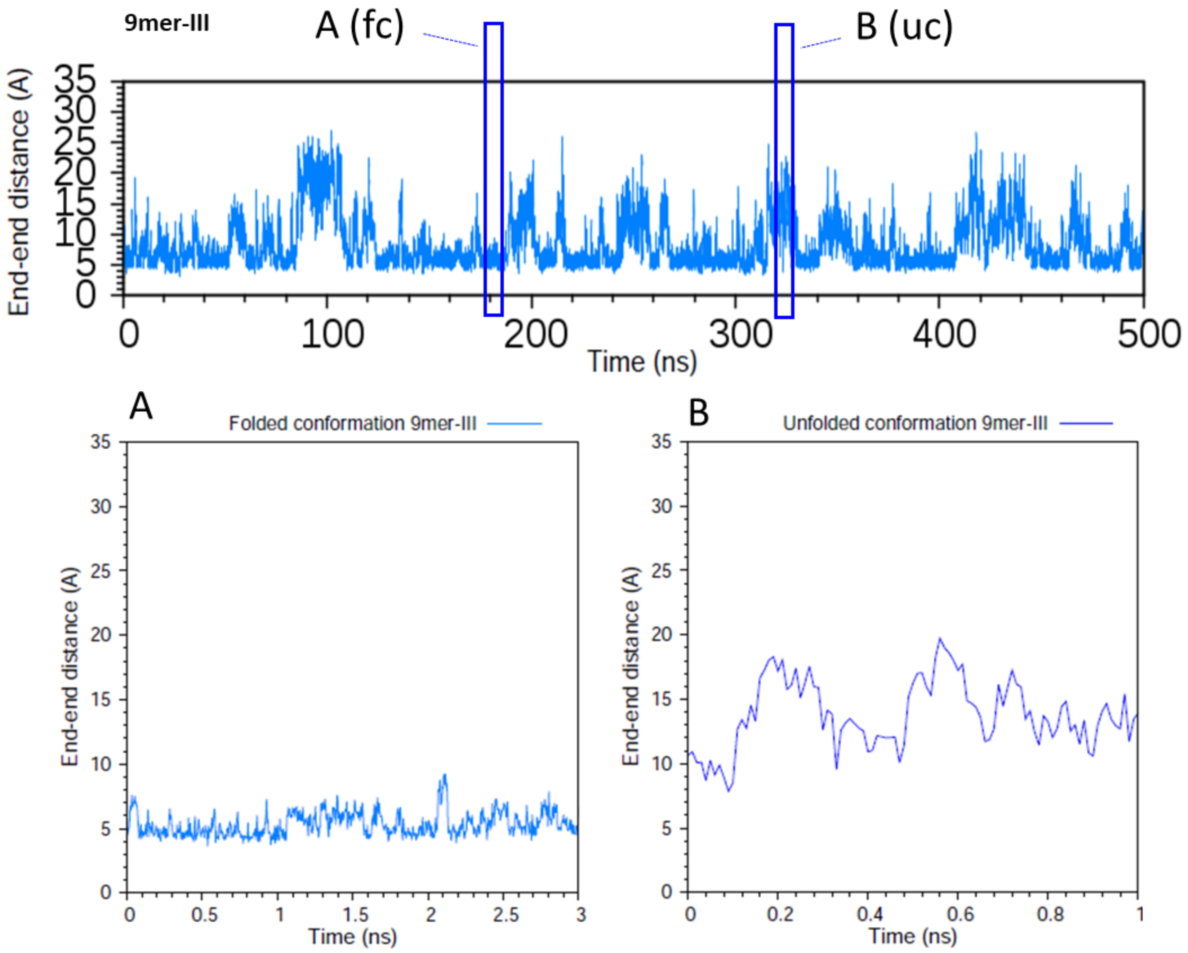

Figure 7 End-to-End Distance of selected intervals of **9mer-III** MD simulations. The top panels indicate the time ranges from the full trajectory used to define interval A (folded conformation model) and interval B (unfolded conformation model). The End-to-End distance was calculated using the C-4 hydroxy group of the non-reducing ends in both oligosaccharides.

2.5 Ramachandran plots[2]

Turn unit (Φ: O$^{5'}$-C$^{1'}$-O^{n}-C^{n}, Ψ: C$^{1'}$-O^{n}-C^{n}-C^{n-1})

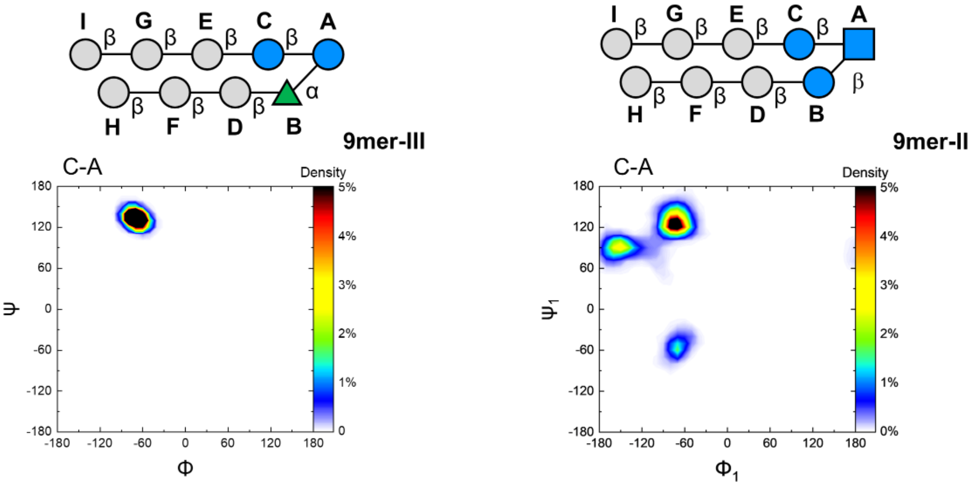

Figure 8 Ramachandran plots of turn unit linkage C-A.

Strand S1 (Φ: H$^{1'}$-C$^{1'}$-O^{4}-C^{4}, Ψ: C$^{1'}$-O^{4}-C^{4}-H^{4})

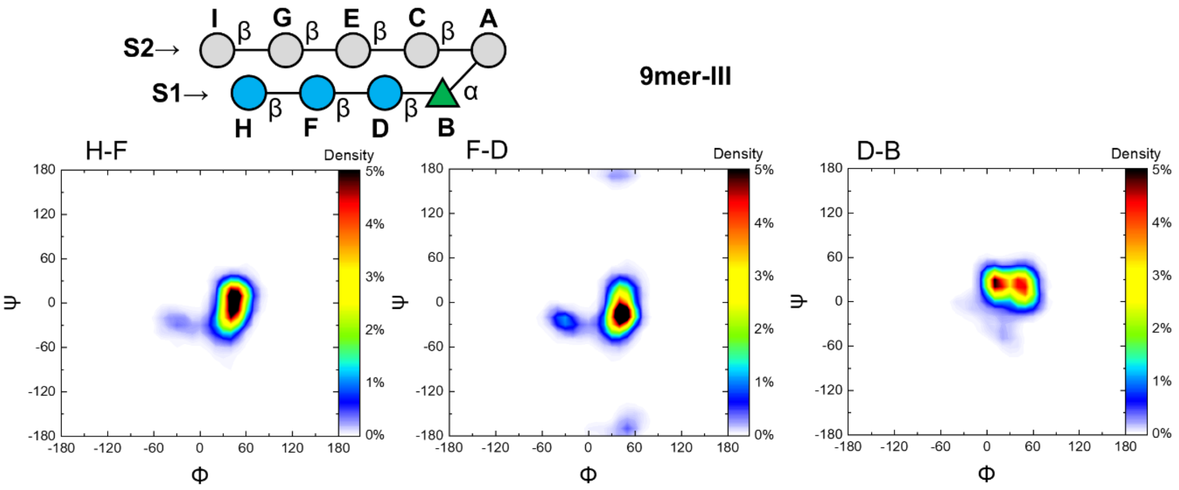

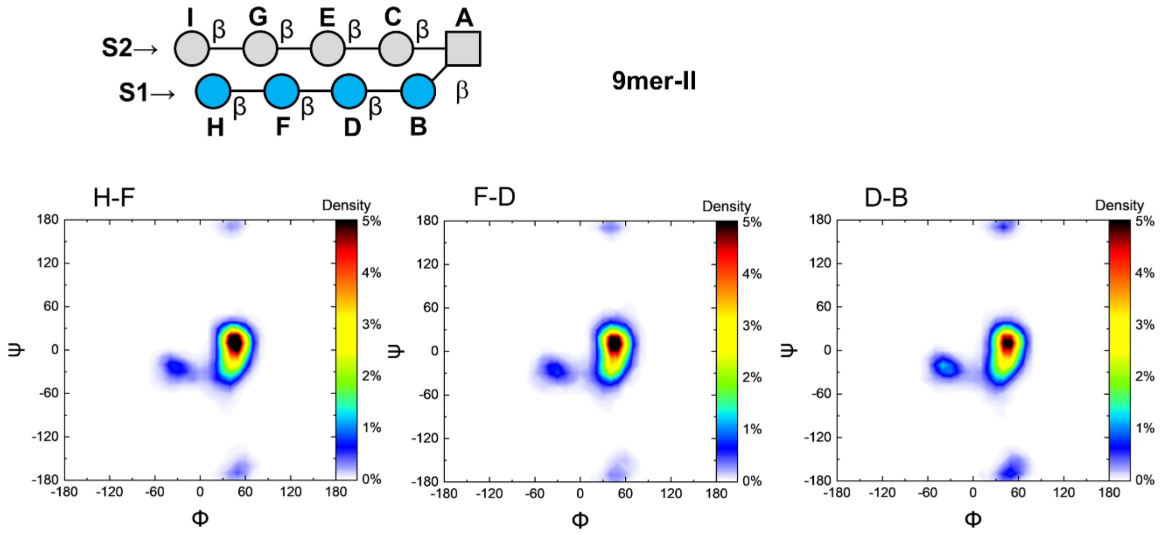

Figure 9 Ramachandran plots of strand S1 linkages.

Strand S2 (Φ: $H^{1'}$-$C^{1'}$-O^{4}-C^{4}, Ψ: $C^{1'}$-O^{4}-C^{4}-H^{4})

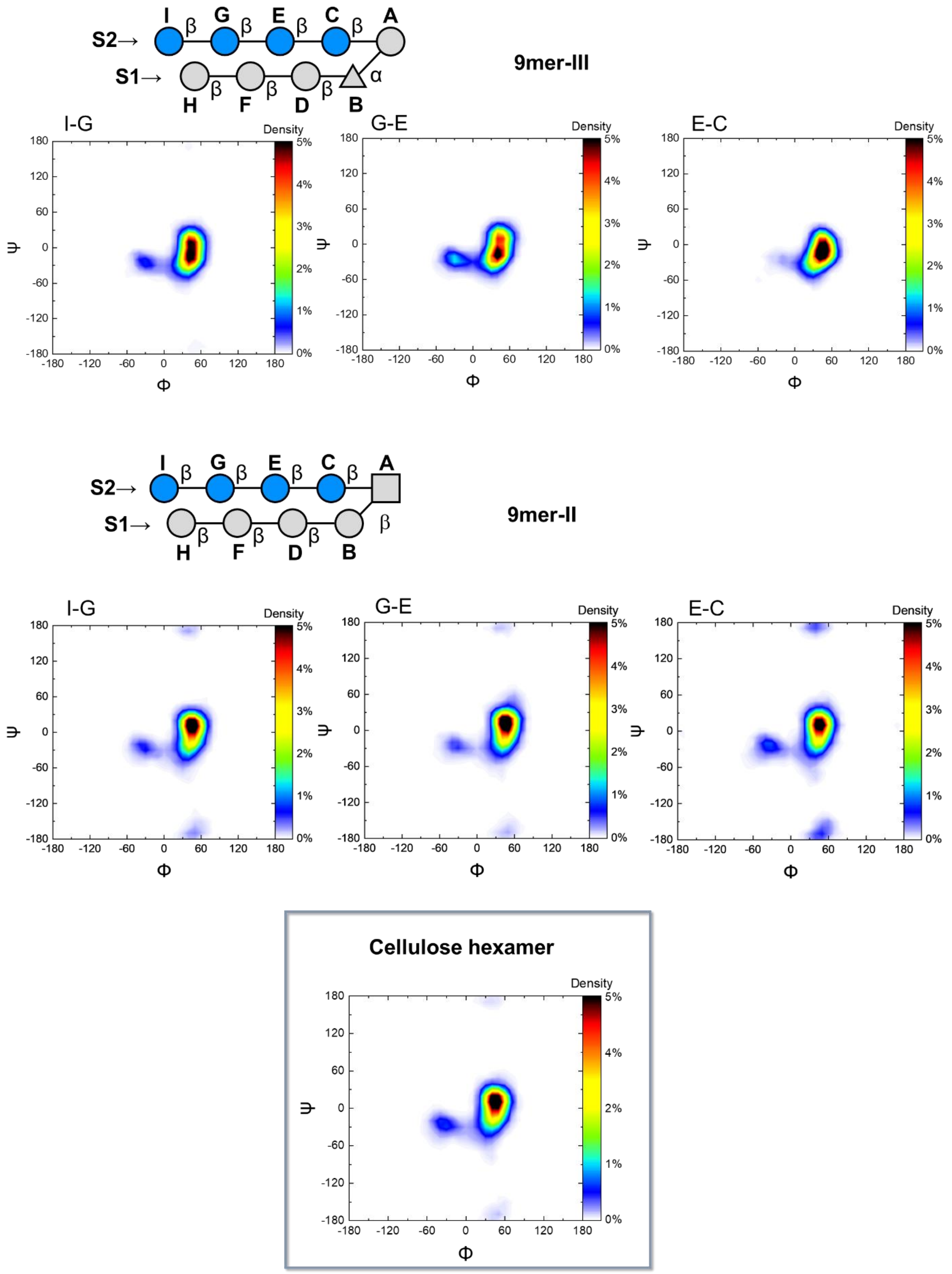

Figure 10 Ramachandran plots of strand S2 linkages and additional Ramachandran plot of glycosidic linkages in cellulose hexamer.

2.6 Intra-strands and inter-strands hydrogen bond pairs extracted from the MD simulation

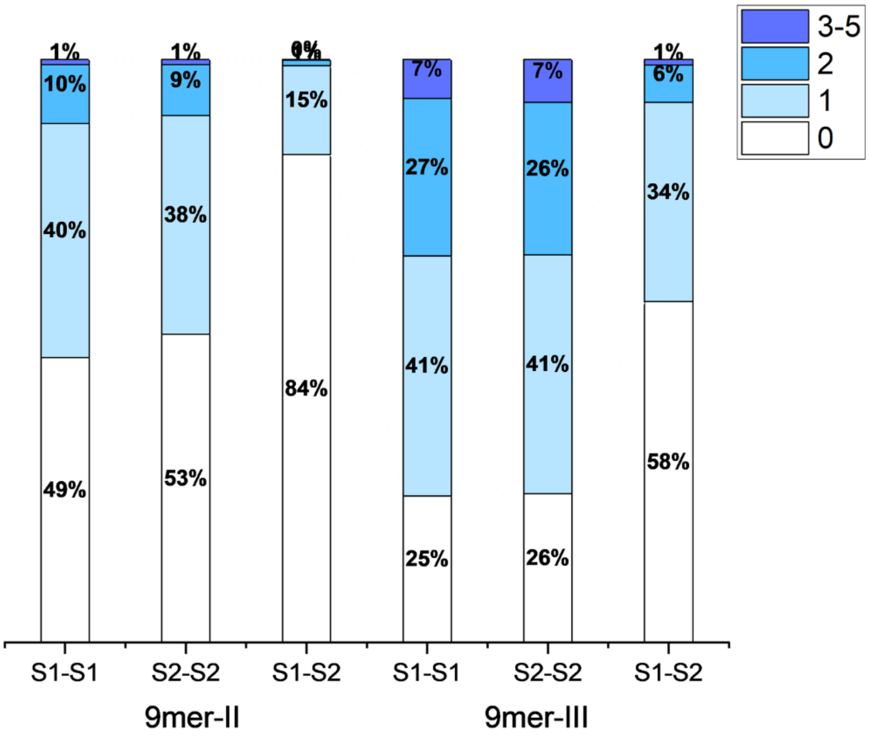

Figure 11 Intra-strands and inter-strands hydrogen bond pairs extracted from the MD simulation.

9mer-II

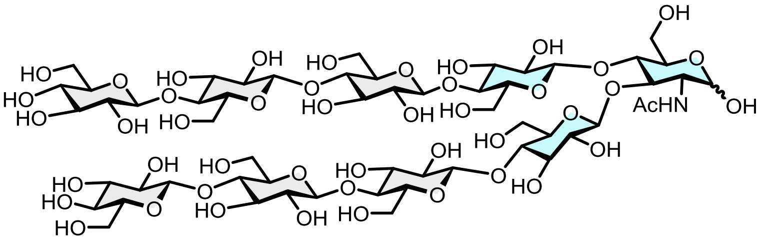

S1-S1

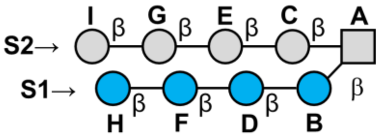

Donor	Acceptor
OH-6, B	O-3, A; O-5, B; O-4, B; OH-2, D; O-5, D; OH-6, D.
OH-3, B	O-3, A; OH-2, D; O-5, D; OH-6, D.
OH-2, B	O-4, B; OH-6, D.
OH-6, D	O-4, B; O-4, D; OH-3, D; OH-2, F; OH-6, F; OH-6, H.
OH-3, D	OH-2, F; O-5, F; OH-6, F.
OH-2, D	O-5, F.

OH-6, F	O-4, F; OH-2, H; O-5, H; OH-6, H.
OH-3, F	OH-2, H; O-5, H; OH-6, H.
OH-6, H	O-4, F; OH-4, H.

S2-S2

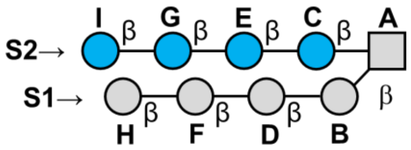

Donor	Acceptor
OH-6, C	O-4, A; OH-3, C; O-4, C; OH-2, E; O-5, E; OH-6, E.
OH-3, C	O-4, A; OH-2, E; O-5, E; OH-6, E.
OH-2, C	O-4, C; O-5, E.
OH-6, E	O-4, C; OH-3, E; OH-2, G; OH-6, G; OH-6, I.
OH-3, E	OH-2, G; O-5, G; OH-6, G.
OH-6, G	O-4, G; OH-2, I; OH-3, I; O-5, I; OH-6, I.
OH-3, G	OH-2, I; O-5, I; OH-6, I.
OH-6, I	OH-4, I.
OH-4, I	OH-2, I.

S1-S2

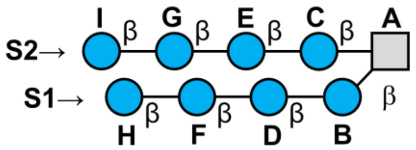

Donor	Acceptor
OH-6, B	O-4, A; OH-3, C; O-4, C; OH-2, C; O-5, C; OH-6, C; O-5, E; OH-2, E; OH-6, E.
OH-3, B	OH-6, C; OH-2, E.
OH-2, B	OH-2, C; OH-6, C; O-5, C.
OH-6, D	OH-6, C; OH-3, E; OH-2, E; OH-2, G.
OH-3, D	OH-3, E; OH-6, E; OH-2, G; OH-6, G.
OH-2, D	OH-6, C; OH-3, C; O-5, E; OH-3, E; OH-6, E; OH-2, G.
OH-6, F	OH-6, C; OH-6, E; OH-3, E; OH-2, E; OH-2, G; OH-3, G; OH-6, G; OH-2, I; OH-6, I.
OH-3, F	OH-2, G; OH-3, G; OH-2, I; OH-3, I; OH-6, I.
OH-2, F	OH-3, E; O-4, E; OH-2, G; OH-3, G; O-5, G; OH-6, G.
OH-6, H	OH-2, G; OH-3, G; OH-6, G; OH-2, I; OH-3, I; OH-4, I; OH-6, I.
OH-4, H	OH-3, I; OH-6, I.
OH-3, H	OH-3, G; OH-6, G; OH-2, I; OH-3, I; OH-6, I.
OH-2, H	OH-3, E; O-4, E; OH-3, G; O-5, G; OH-3, I; OH-4, I; OH-6, I.
OH-6, C	O-3, A; O-5, B.
OH-2, C	O-3, A; O-5, B; OH-6, D.
OH-6, E	OH-3, B, O-4, B; O-4, D; OH-6, D; O-5, F; OH-2, H.

OH-3, E	O-4, D.
OH-2, E	OH-2, D.
OH-6, G	O-4, D; OH-3, F; O-5, F; OH-4, H.
OH-3, G	OH-3, D; O-4, D; O-5, H; OH-4, H.
OH-2, G	O-4, D; O-5, F; OH-2, H.
OH-4, I	OH-4, H.
OH-2, I	OH-2, H; OH-4, H.

9mer-III

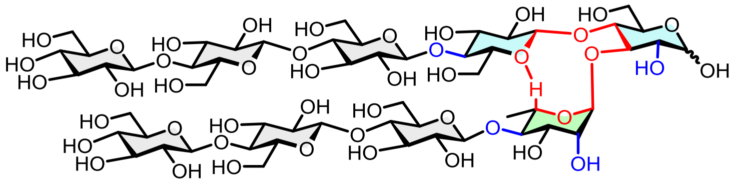

S1-S1

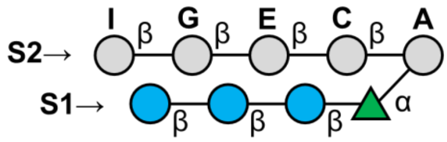

Donor	Acceptor
OH-6, B	OH-2, D; O-5, D.
OH-6, D	O-4, D; OH-2, F; O-5, F; OH-6, H.
OH-3, D	OH-2, F; O-5, F; OH-6, F.
OH-6, F	O-4, D; O-4, F; OH-2, H; OH-3, H.
OH-3, F	O-5, H; OH-6, H.
OH-6, H	O-4, F; OH-4, H.
OH-4, H	OH-2, H.

S2-S2

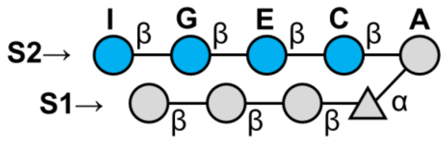

Donor	Acceptor
OH-6, C	O-4, C; OH-2, E.
OH-3, C	O-4, A; OH-2, E; O-5, E; OH-6, E.
OH-6, E	O-4, E; OH-2, G.
OH-3, E	O-5, G; OH-6, G.
OH-6, G	O-4, G; OH-2, I; OH-6, I.

OH-3, G	OH-2, I; O-5, I; OH-6, I.
OH-6, I	O-4, G; OH-4, I.

S1-S2

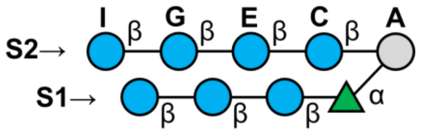

Donor	Acceptor
OH-3, B	OH-6, C; OH-2, E.
OH-6, D	OH-3, C; OH-2, C; OH-3, E; OH-2, E; O-4, E; OH-6, E; OH-2, G.
OH-3, D	OH-3, C; OH-2, E; OH-3, E; OH-6, E; OH-2, G; OH-6, G.
OH-2, D	OH-6, C; OH-3, C; O-5, E; OH-2, E; OH-6, E.
OH-6, F	OH-6, E; OH-3, E; O-5, E; OH-2, E; OH-2, G; OH-3, G; O-5, G; OH-6, G; OH-2, I.
OH-3, F	OH-3, G; OH-2, I; OH-6, I.
OH-2, F	OH-6, E; O-4, E; OH-2, G; OH-3.
OH-6, H	OH-2, G; OH-3, G; OH-6, G; OH-4, I; OH-6, I.
OH-4, H	OH-2, I; OH-6, I.
OH-3, H	OH-3, G; OH-6, G; OH-2, I; OH-4, I; O-5, I; OH-6, I.
OH-2, H	O-4, E; OH-3, G; OH-2, G; OH-6, G; OH-2, I; OH-4, I; O-5, I; OH-6, I.
OH-6, C	O-4, B.
OH-3, C	O-5, D.
OH-6, E	O-5, D; O-5, F.
OH-6, G	O-4, F; O-5, F.
OH-3, G	O-4, F; O-5, H.
OH-2, G	OH-3, D; O-4, D; OH-3, F; O-4, F; O-5, H.
OH-6, I	O-5, H.
OH-3, I	OH-3, H; OH-4, H.
OH-2, I	O-4, F; OH-6, H.

2.7 Comparison of RMSD, End-to-End Distance and Radius of Gyration profiles of 9mer-II for 3 replicates

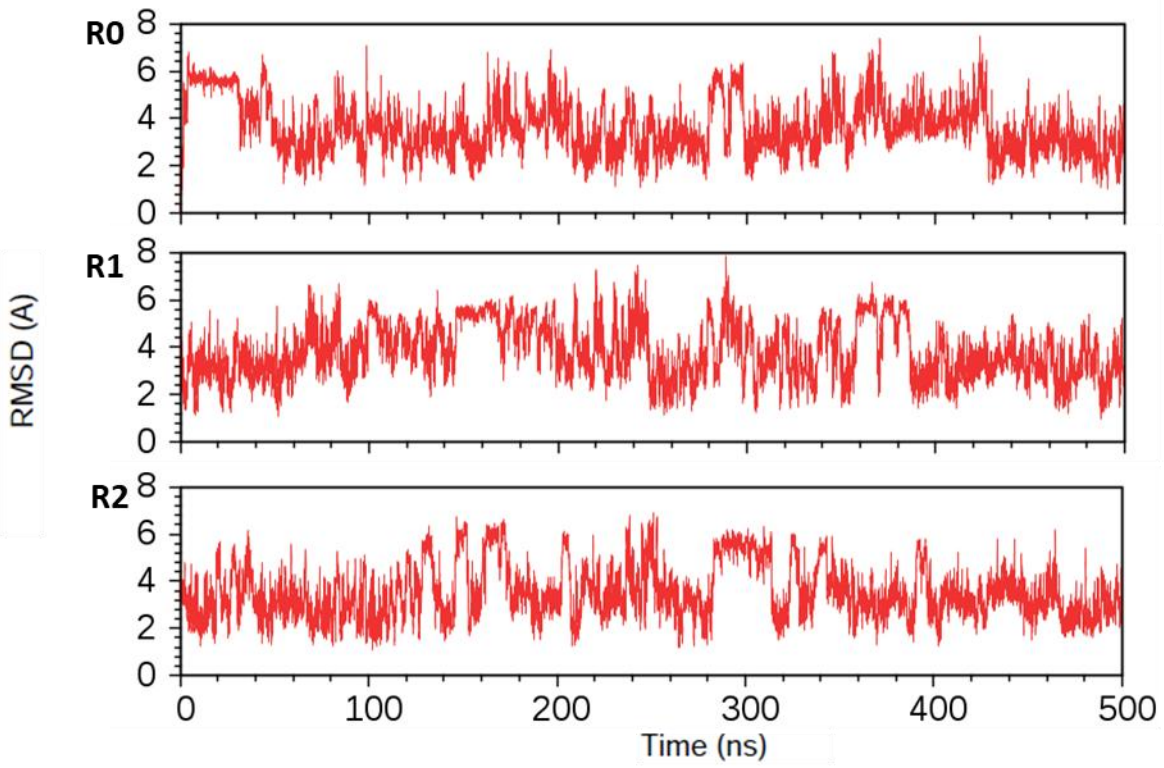

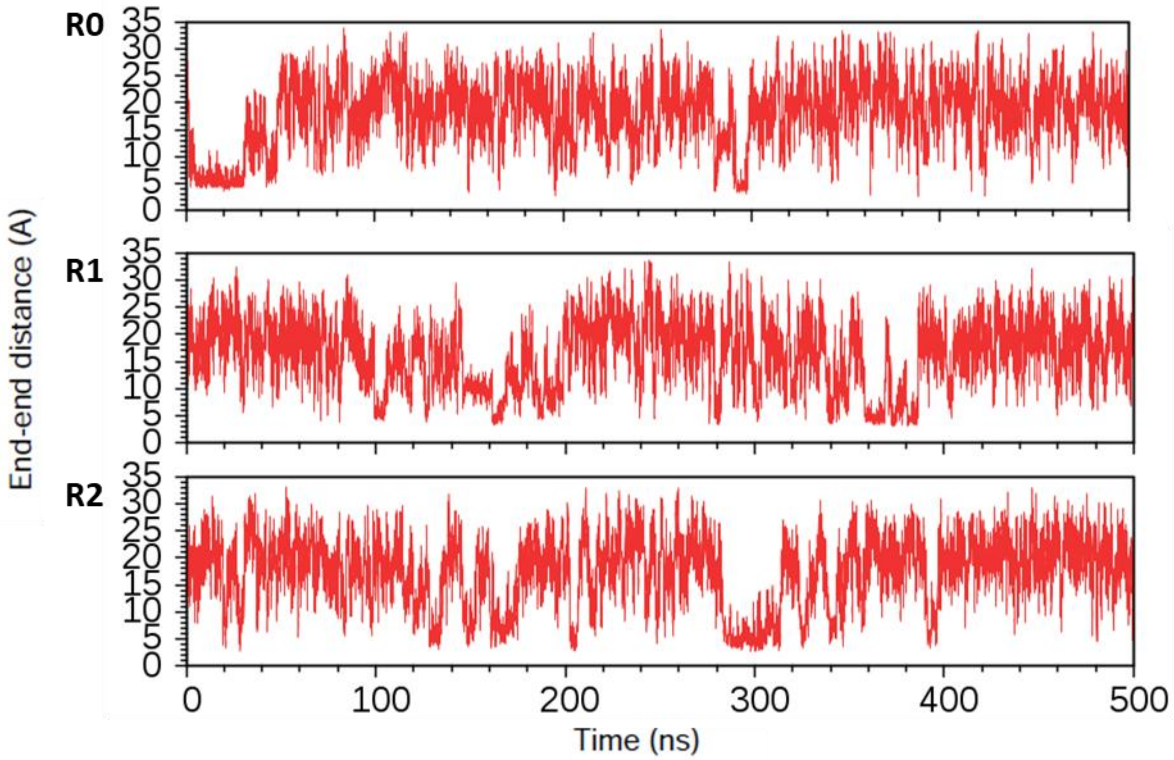

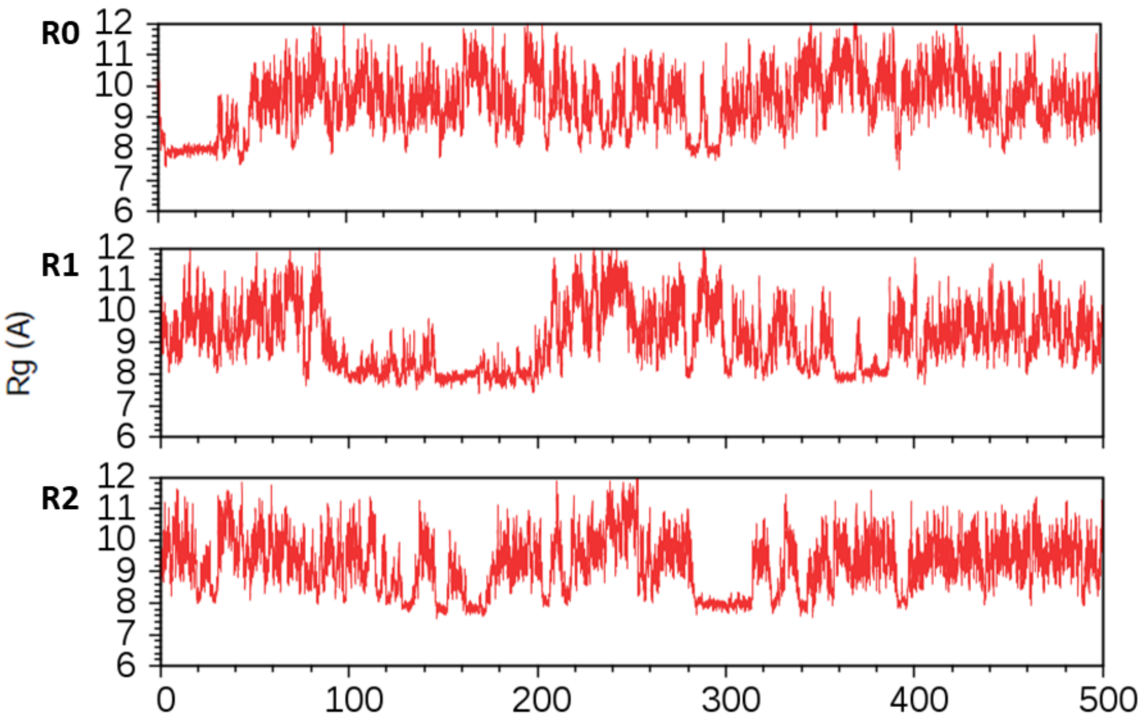

Figure 12 Comparison of RMSD, End-to-End Distance and Radius of Gyration profiles of **9mer-II** between the original MD simulation and two replicate runs initialized with different velocity seeds, showing the reproducibility of structural dynamics. The End-to-End distance was calculated using the C-4 hydroxy group of the non-reducing ends in both oligosaccharides.

2.8 Comparison of RMSD, End-to-End Distance and Radius of Gyration profiles of 9mer-III for 3 replicates

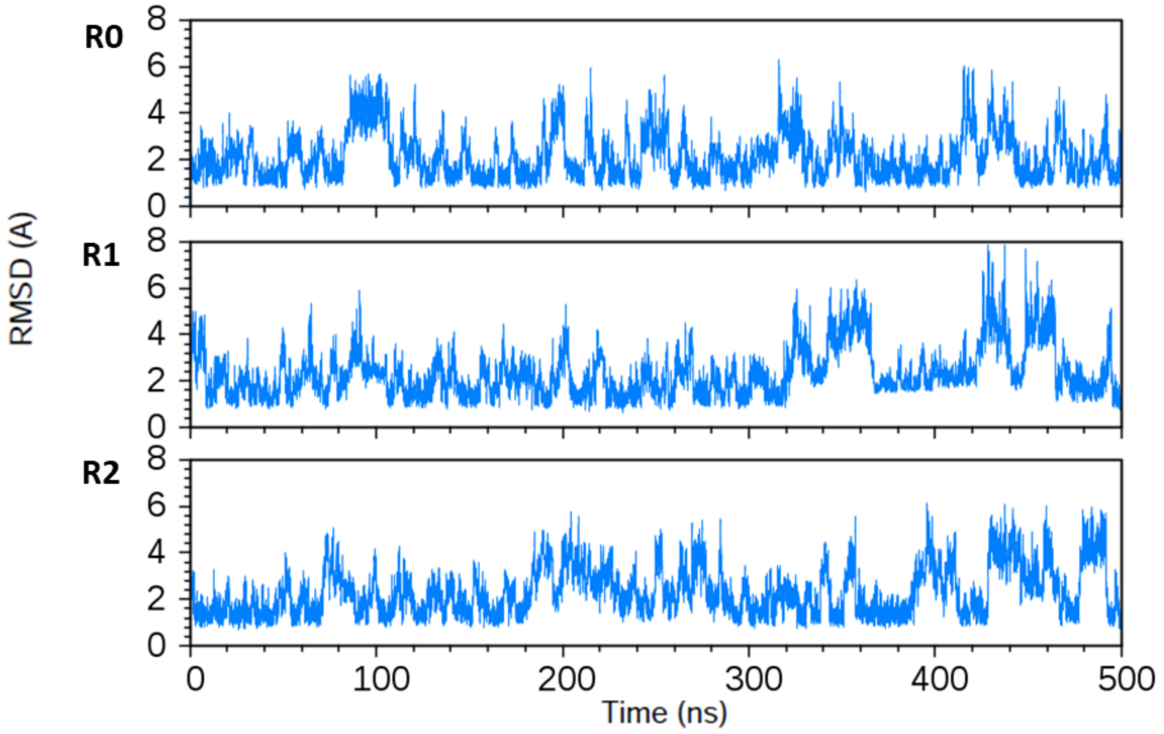

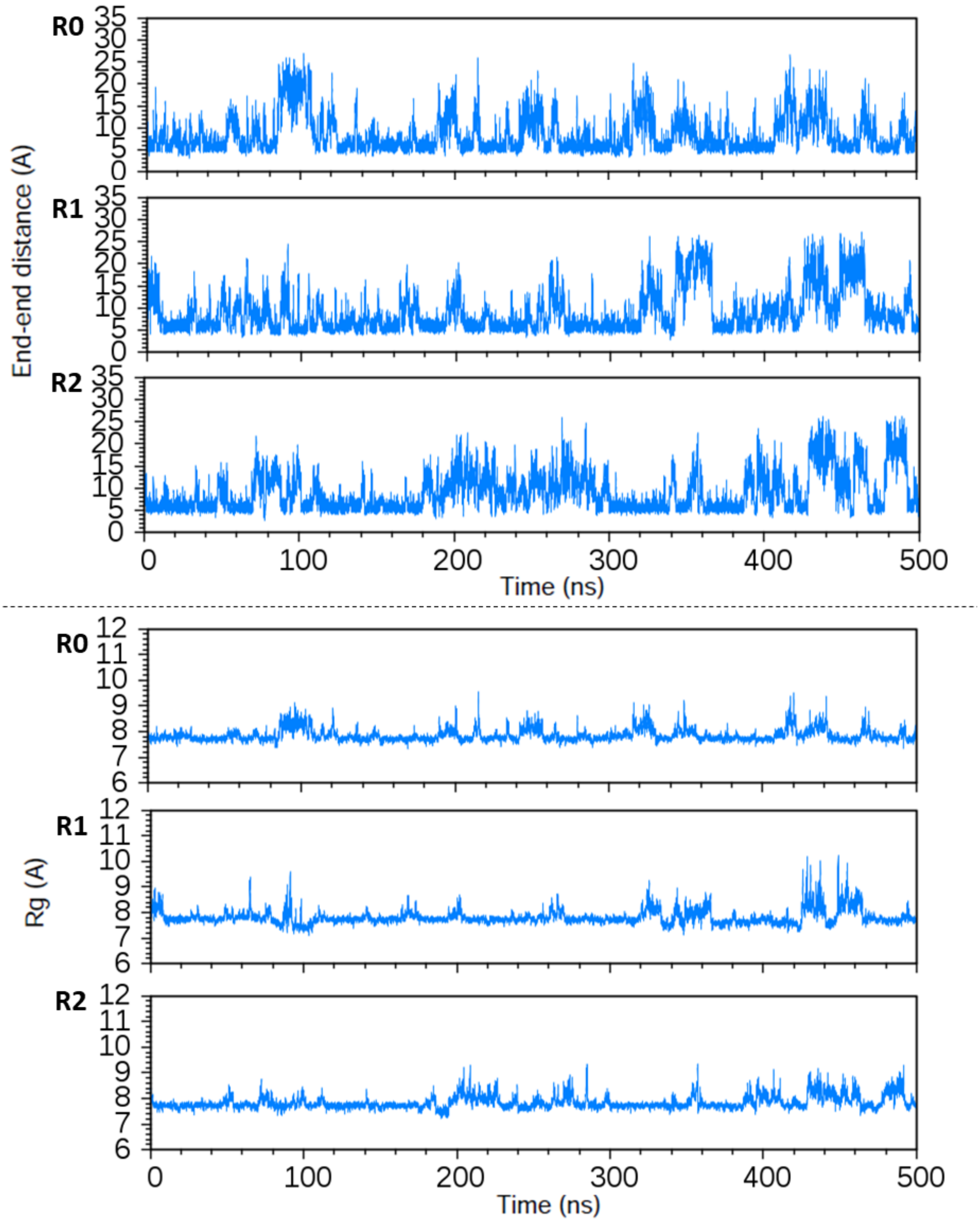

Figure 13 Comparison of RMSD, End-to-End Distance and Radius of Gyration profiles of **9mer-III** between the original MD simulation and two replicate runs initialized with different velocity seeds, showing the reproducibility of structural dynamics. The End-to-End distance was calculated using the C-4 hydroxy group of the non-reducing ends in both oligosaccharides.

2.9 Comparison of Inter-residue Distance of 9mer-II between center of mass of homologous residues in both strands

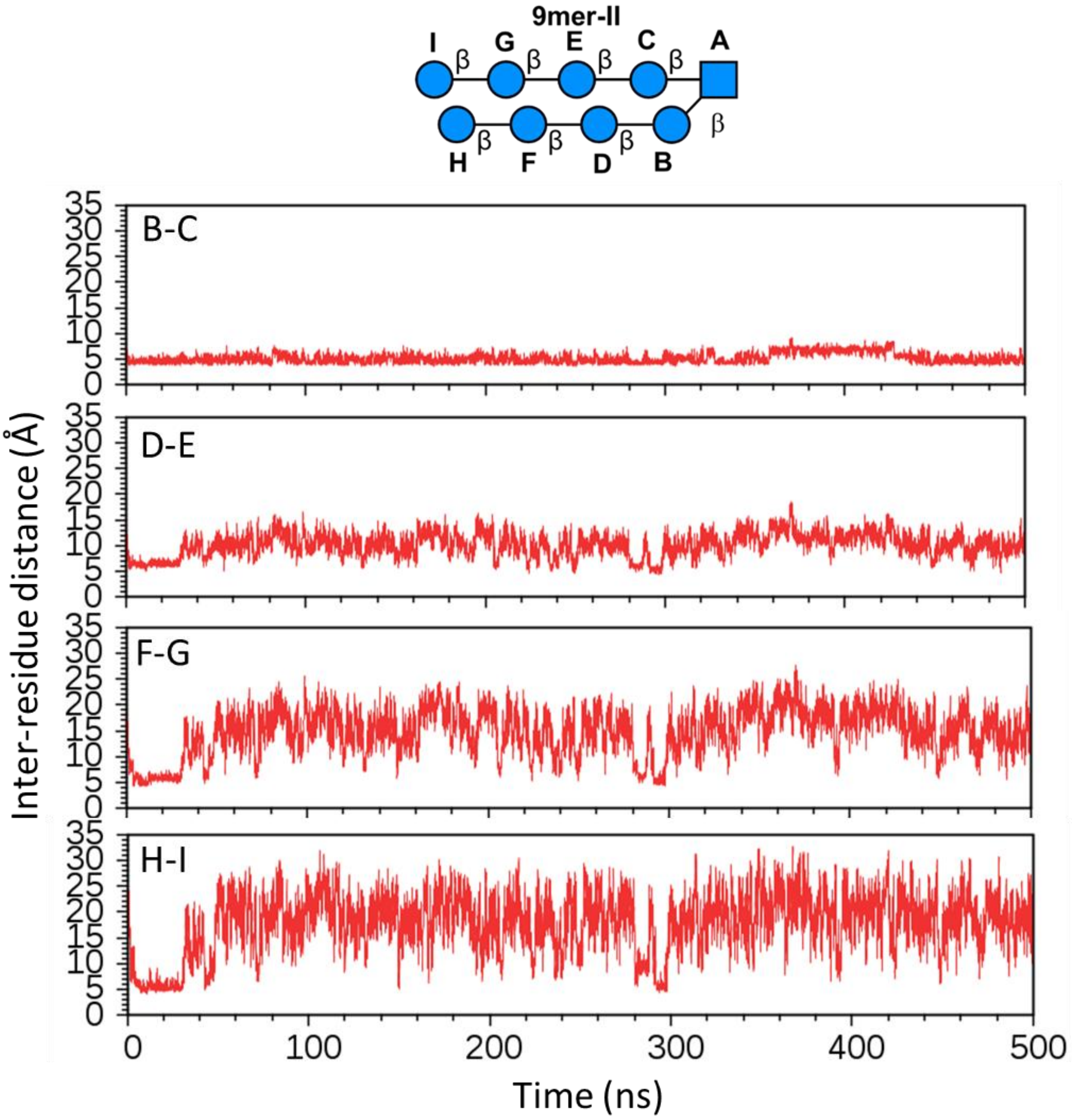

Figure 14 Comparison of Inter-residue Distance of **9mer-II** between center of mass of homologous residues in both strands.

2.10 Comparison of Inter-residue Distance of 9mer-III between center of mass of homologous residues in both strands

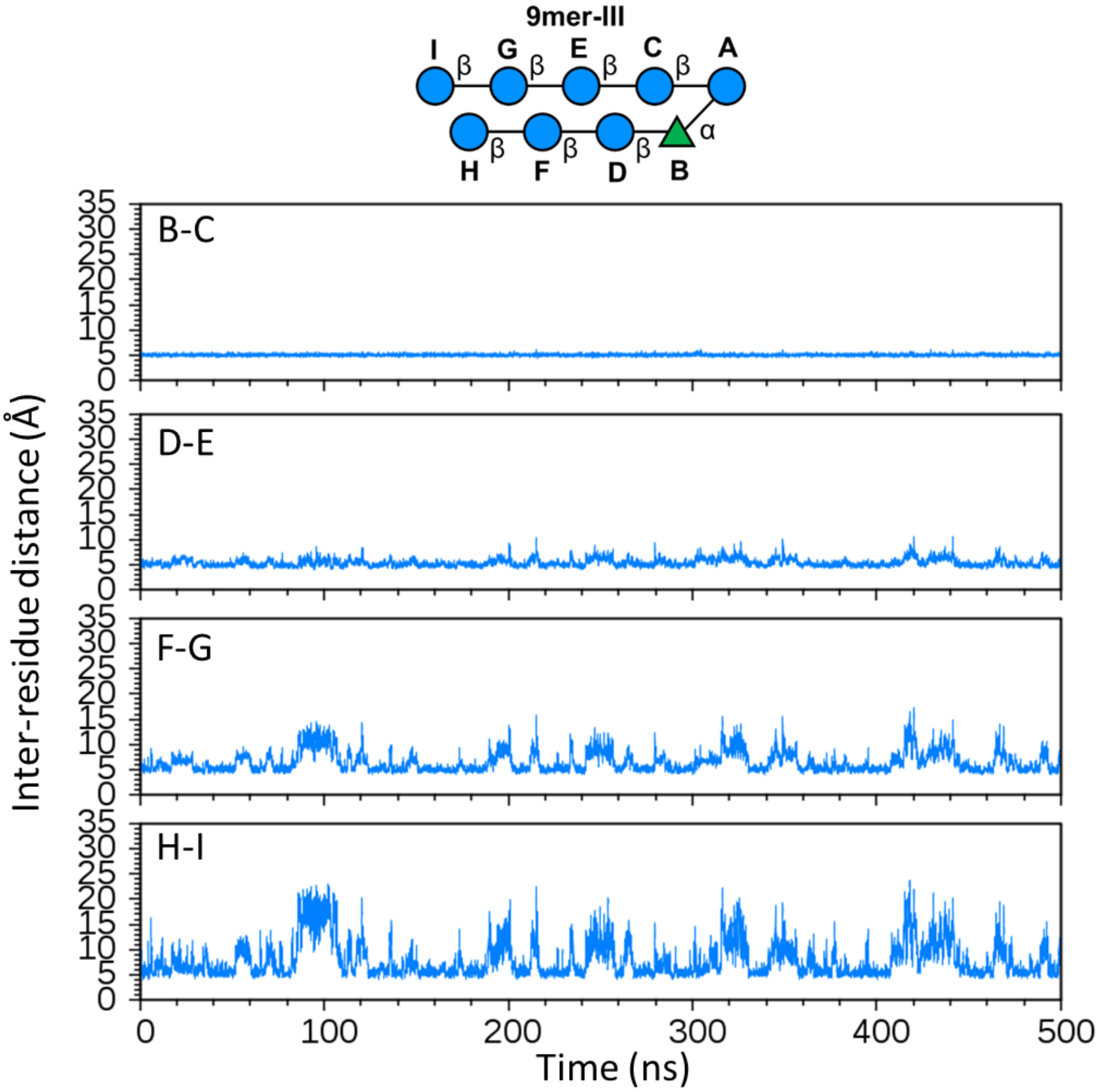

Figure 15 Comparison of Inter-residue Distance of **9mer-III** between center of mass of homologous residues in both strands.

3 SAXS calculation

3.1 General materials and methods

Explicit-solvent SAXS calculations were performed based on a modified version[14,15] of GROMACS 2022.2 (GROMACS-SWAXS)[16]. Details of explicit-solvent SAXS calculations are presented in a previous publication.[17] Here, in this modified Gromacs code, the solvent molecules in the solvation layer were considered in the SAXS intensity calculation, in contrast to the common approach where only the solute molecules are used for the SAXS intensity simulations. This explicit solvent model can provide realistic simulated intensities, as the density fluctuation of water molecules around the solute certainly contributes to the overall SAXS intensities. A spatial envelope was built around the hairpin at a distance of 0.5, 0.7 and 0.9 nm, among which all the water molecules contribute to the SAXS intensities. The water subtraction was carried out using 100 simulation frames of the pure-water simulation box, which was simulated for 10 ns and which was large enough to enclose the envelope. The atomic form factors were approximated by $f(q) = c + \sum_{k=1}^{4} a_k \exp[-b_k(q/4\pi)^2]$, where the values a_k, b_k and c are the Cromer-Mann parameters[18]. The orientational average was carried out using 500 q-vectors for each absolute value of q, and the solvent electron density was corrected to the experimental value of 334 e/nm^3.

3.2 Calculated SAXS intensity curves of two representative conformations of 9mer-II and 9mer-III using different envelopes

9mer-II

- Envelope: 0.2 nm

fc

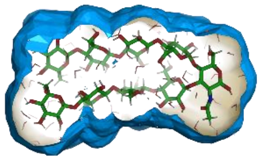

uc

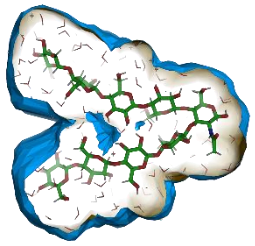

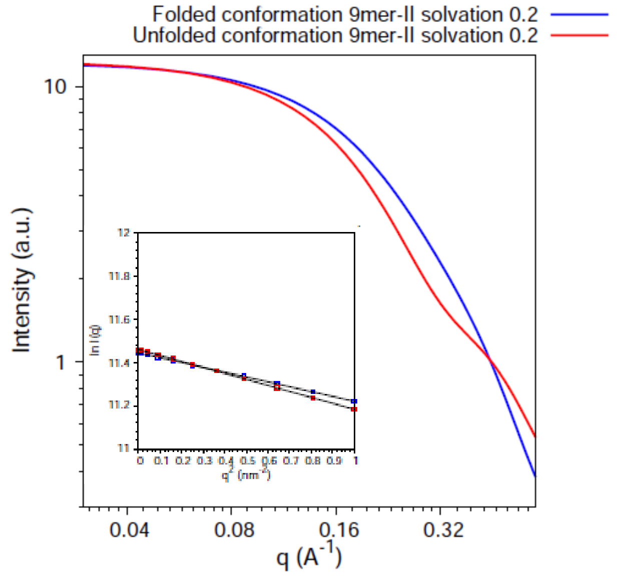

Table of curve fitting of natural logarithm of I(q) vs. q^2 plots of Guinier law-SAXS measurements of calculated SAXS curves.

	Folded conformation (fc)		Unfolded conformation (uc)	
	**Value**	**Error**	**Value**	**Error**
**Slope**	-0.2241	0.0005	-0.2773	0.0003
**I_0**	11.44	0.0002	11.46	0.0001
**R_g (Å)**	8.200	0.009	9.122	0.005
**RMSE**	0.00054		0.00032	

- Envelope: 0.3 nm

fc

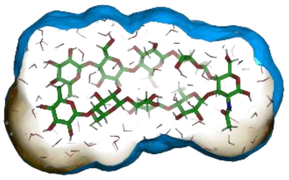

uc

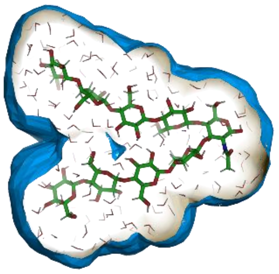

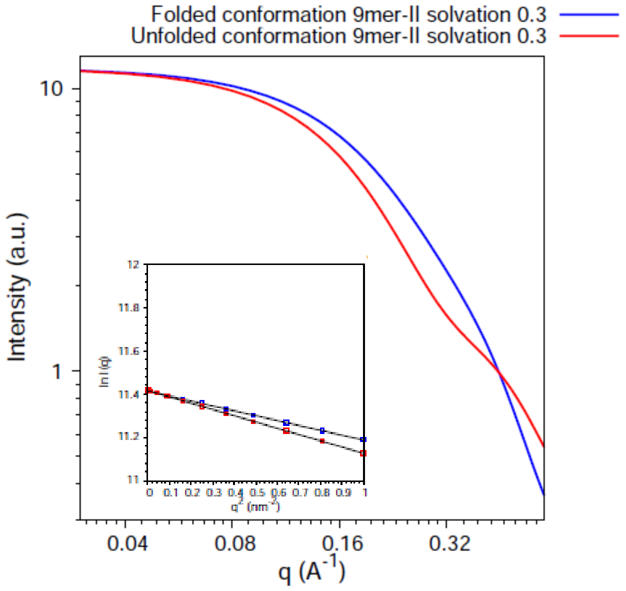

Table of curve fitting of natural logarithm of I(q) vs. q^2 plots of Guinier law-SAXS measurements of calculated SAXS curves.

	Folded conformation (fc)		Unfolded conformation (uc)	

	Value	Error	Value	Error
**Slope**	-0.2263	0.0005	-0.2906	0.0003
**I_0**	11.41	0.0003	11.42	0.0002
**R_g (Å)**	8.234	0.010	9.336	0.006
**RMSE**	0.00057		0.00043	

- Envelope: 0.5 nm

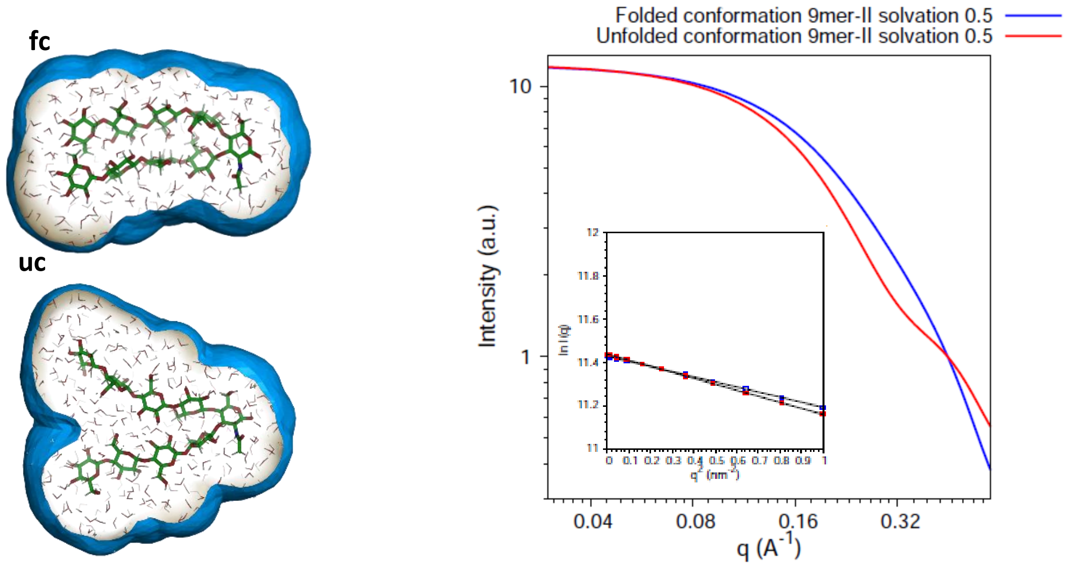

Table of curve fitting of natural logarithm of I(q) vs. q^2 plots of Guinier law-SAXS measurements of calculated SAXS curves.

	Folded conformation (fc)		Unfolded conformation (uc)	
	**Value**	**Error**	**Value**	**Error**
**Slope**	-0.2360	0.0006	-0.2787	0.0001
**I_0**	11.43	0.0003	11.44	0.0001
**R_g (Å)**	8.415	0.011	9.143	0.002
**RMSE**	0.00068		0.00011	

- Envelope: 0.7 nm

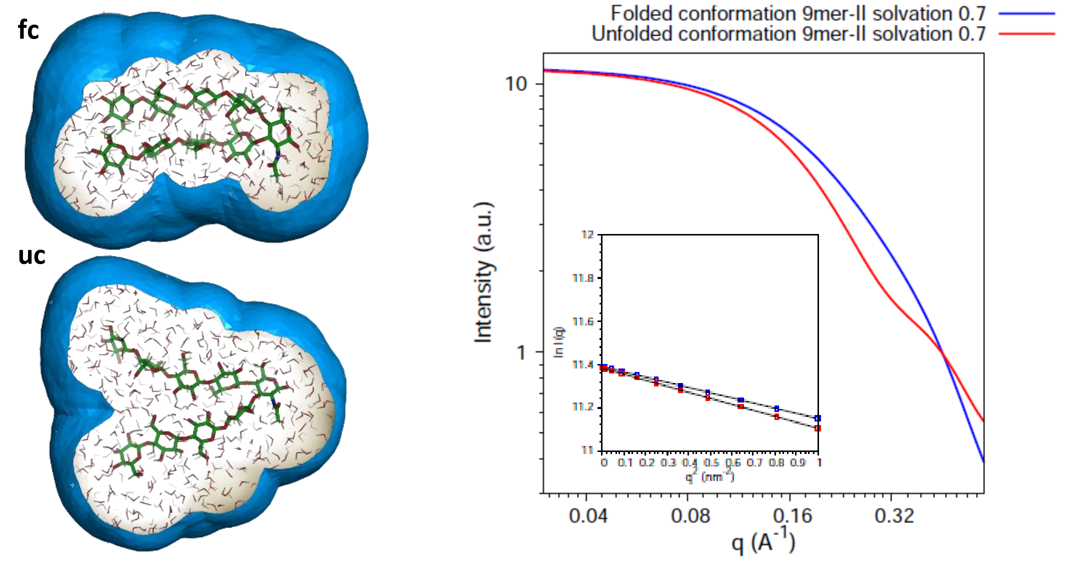

Table of curve fitting of natural logarithm of I(q) vs. q^2 plots of Guinier law-SAXS measurements of calculated SAXS curves.

Folded conformation (fc) **Unfolded conformation (uc)**

	Value	Error	Value	Error
**Slope**	-0.2401	0.0011	-0.2783	0.0002
**I_0**	11.39	0.0005	11.38	0.0001
**R_g (Å)**	8.487	0.019	9.138	0.004
**RMSE**	0.00118		0.00020	

- Envelope: 0.9 nm

fc

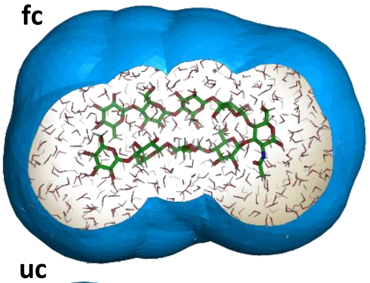

uc

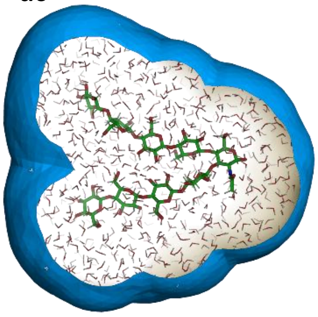

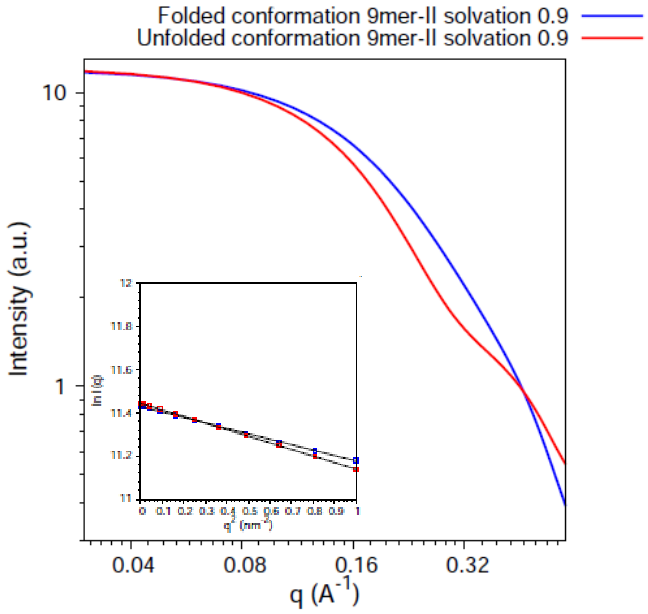

Table of curve fitting of natural logarithm of I(q) vs. q^2 plots of Guinier law-SAXS measurements of calculated SAXS curves.

	Folded conformation (fc)		Unfolded conformation (uc)	
	**Value**	**Error**	**Value**	**Error**
**Slope**	-0.2520	0.0014	-0.3055	0.0005
**I_0**	11.43	0.0007	11.44	0.0003
**R_g (Å)**	8.695	0.024	9.574	0.008
**RMSE**	0.00150		0.00057	

- Envelope: 1.0 nm

fc

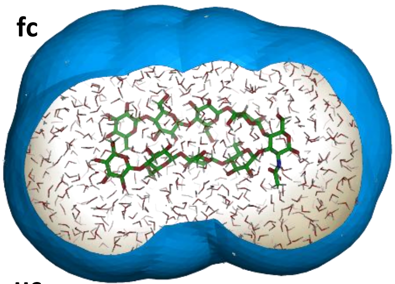

uc

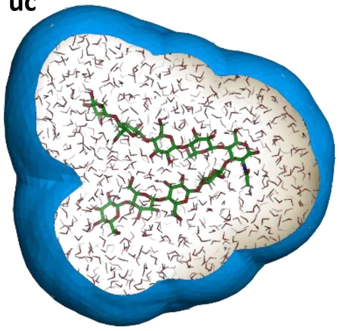

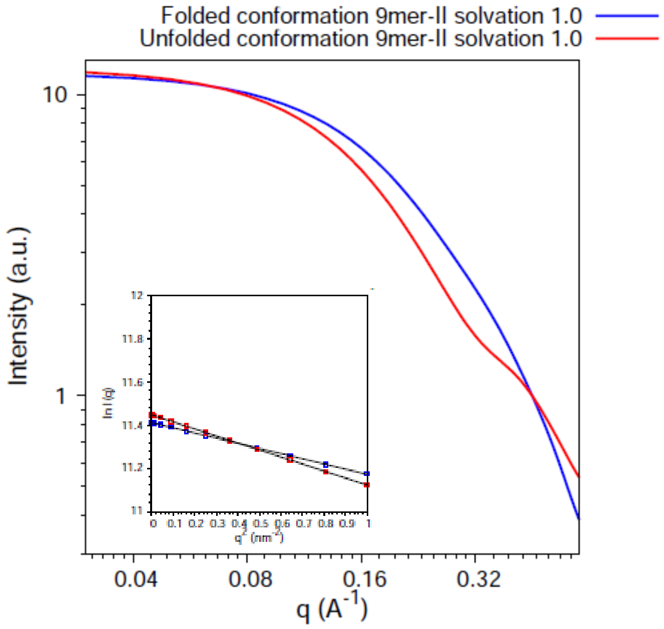

Table of curve fitting of natural logarithm of I(q) vs. q^2 plots of Guinier law-SAXS measurements of calculated SAXS curves.

	Folded conformation (fc)		Unfolded conformation (uc)	
	**Value**	**Error**	**Value**	**Error**
**Slope**	-0.2398	0.0010	-0.3252	0.0010
$\mathbf{I_0}$	11.41	0.0005	11.45	0.0005
$\mathbf{R_g}$ **(Å)**	8.482	0.018	9.878	0.015
**RMSE**	0.00112		0.00111	

9mer-III

- Envelope: 0.2 nm

fc

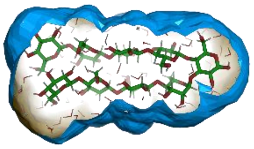

uc

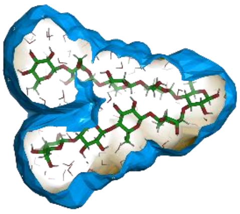

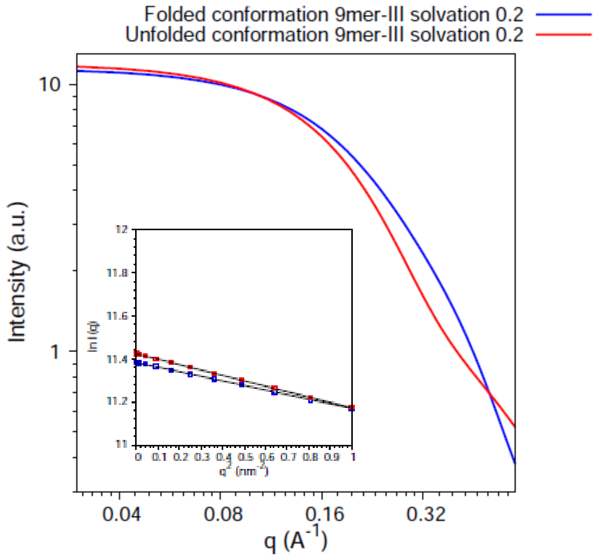

	Folded conformation (fc)		Unfolded conformation (uc)	
	**Value**	**Error**	**Value**	**Error**
**Slope**	-0.2132	0.0005	-0.2512	0.0002
$\mathbf{I_0}$	11.38	0.0002	11.42	0.0001
$\mathbf{R_g}$ **(Å)**	7.998	0.009	8.681	0.004
**RMSE**	0.00055		0.00018	

- Envelope: 0.3 nm

fc

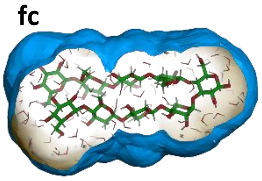

uc

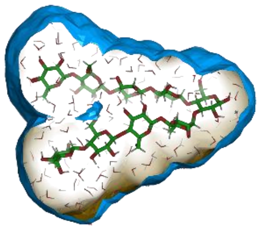

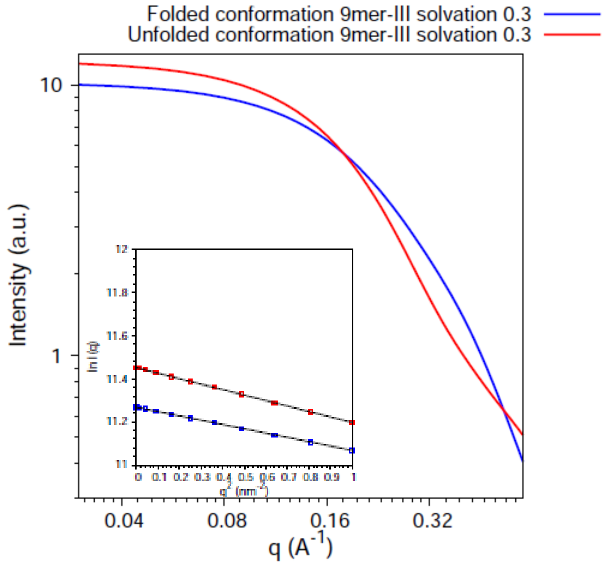

	Folded conformation (fc)		Unfolded conformation (uc)	
	**Value**	**Error**	**Value**	**Error**
**Slope**	-0.1998	0.0004	-0.2534	0.0002
**I_0**	11.27	0.0002	11.45	0.0001
**R_g (Å)**	7.743	0.008	8.720	0.004
**RMSE**	0.00045		0.00024	

- Envelope: 0.5 nm

fc

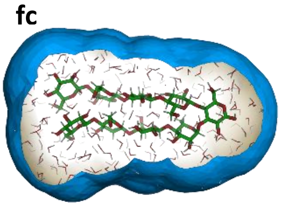

uc

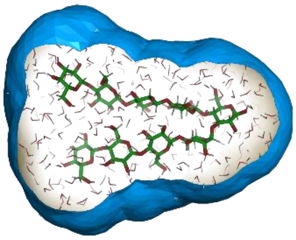

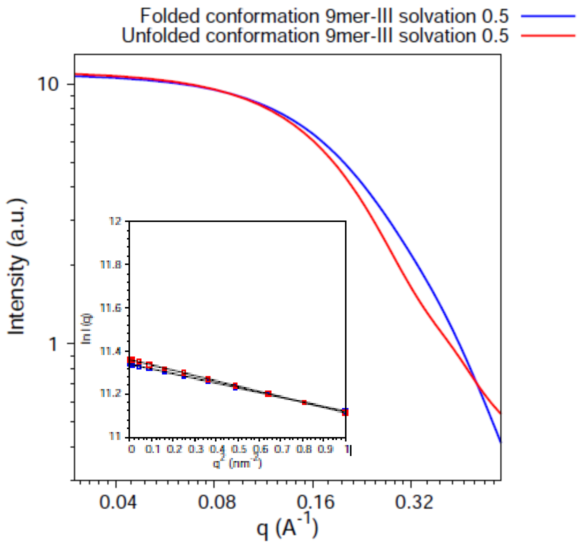

	Folded conformation (fc)		Unfolded conformation (uc)	
	**Value**	**Error**	**Value**	**Error**
**m**	-0.2187	0.0005	-0.2459	0.0004
**I_0**	11.34	0.0003	11.36	0.0002
**R_g (Å)**	8.100	0.010	8.590	0.006
**RMSE**	0.00060		0.00040	

- Envelope: 0.7 nm

fc

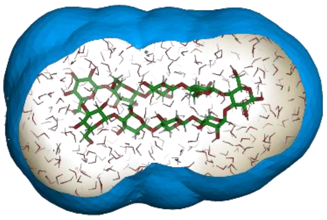

uc

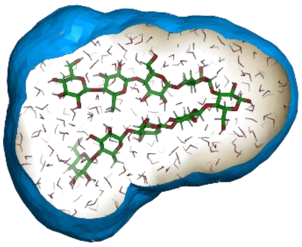

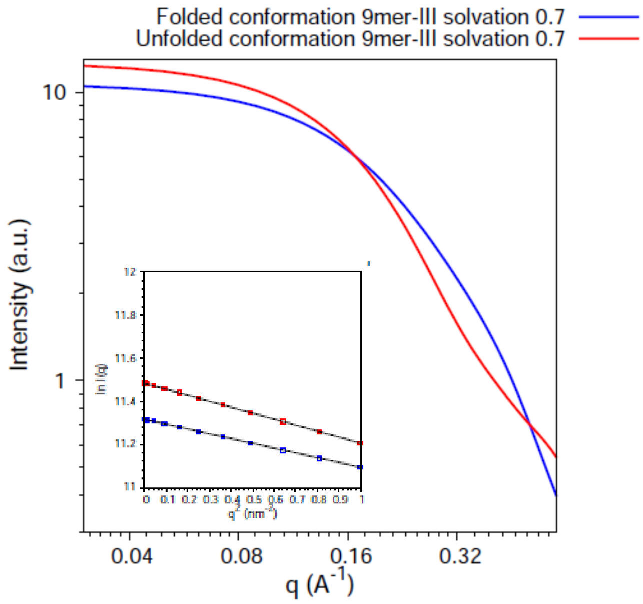

	Folded conformation (fc)		Unfolded conformation (uc)	
	**Value**	**Error**	**Value**	**Error**
**m**	-0.2222	0.0008	-0.2775	0.0005
I_0	11.32	0.0004	11.49	0.0004
R_g (Å)	8.164	0.014	9.124	0.008
**RMSE**	0.00069		0.00056	

- Envelope: 0.9

fc

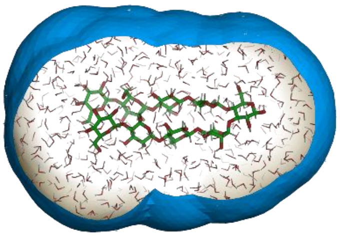

uc

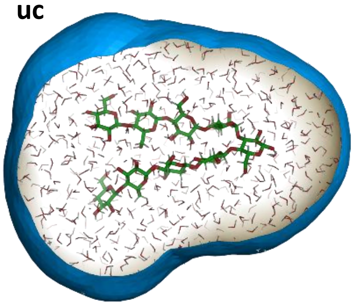

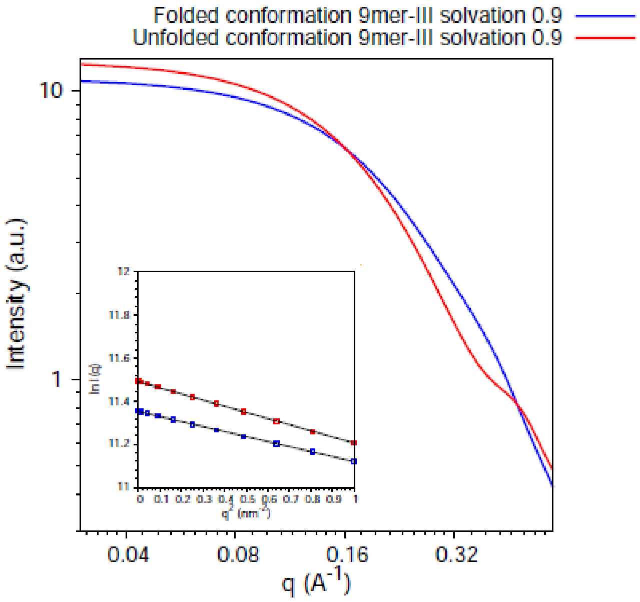

nm

	Folded conformation (fc)		Unfolded conformation (uc)	
	**Value**	**Error**	**Value**	**Error**
**m**	-0.2322	0.0011	-0.2850	0.0008
I_0	11.35	0.0004	11.49	0.0004
R_g (Å)	8.347	0.019	9.246	
**RMSE**	0.00116		0.00087	

- Envelope: 1.0 nm

fc

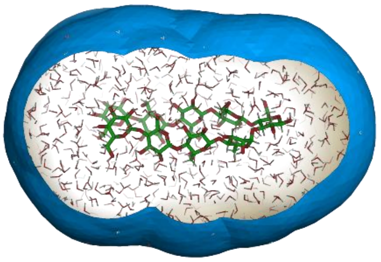

uc

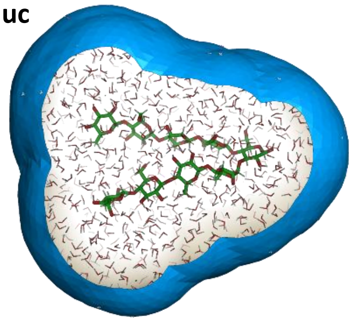

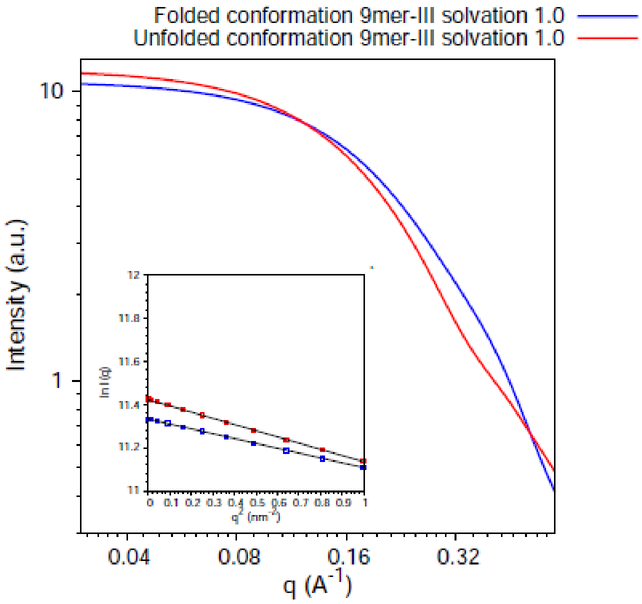

	Folded conformation (fc)		Unfolded conformation (uc)	
	**Value**	**Error**	**Value**	**Error**
**m**	-0.2250	0.0010	-0.2875	0.0012
**n (I_0)**	11.33	0.0005	11.42	0.0006
**R_g (Å)**	8.215	0.018	9.288	0.019
**RMSE**	0.00110		0.00128	

3.3 Radius of gyration and intensity at q=0 vs. solvation layer.

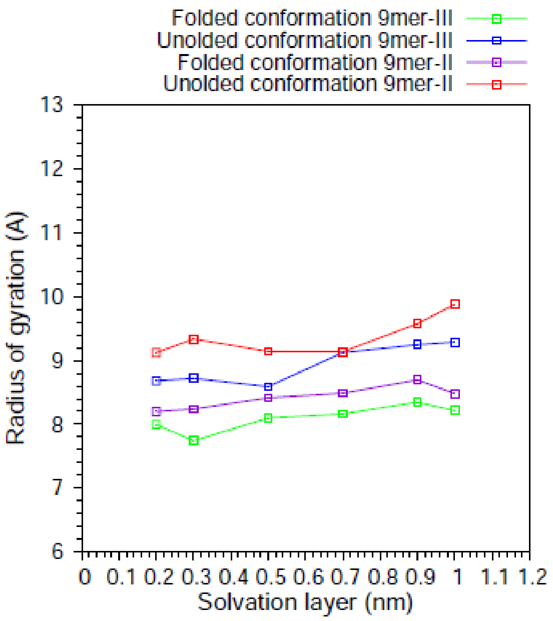

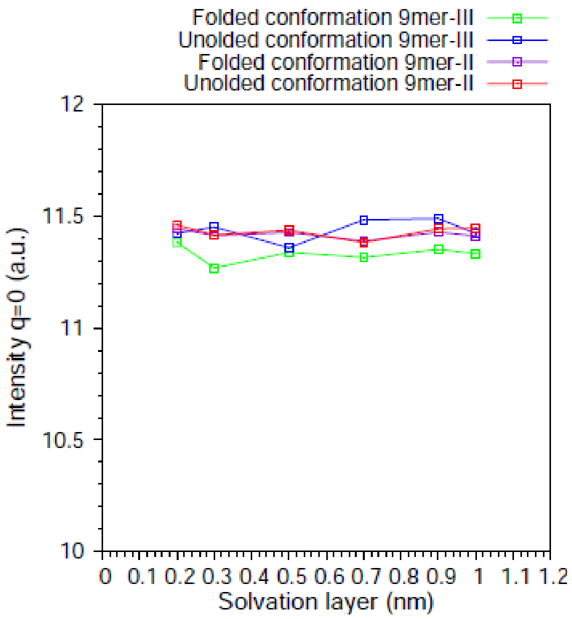

3.4 Analysis of the solvation layer

The distance d of the envelope from the solute atoms must be sufficiently large to ensure bulk-like water state at the envelope surface. If d is chosen too small, density modulations due to the first and second solvation layer may systematically bias the calculated profiles. On the other hand, an unnecessarily large d adds noise to the profiles, because the intensities of the solute system (including its solvation layer) and the excluded solvent rapidly increase with d, whereas the excess intensity I(q) should converge with increasing d.[14]

Radial pair distribution functions (RDF) g(r), were calculated between center-of-mass of selected residues of the glycan hairpins and all of the water molecules in order to characterize the solvation around some parts of the molecule. The distribution function gives the probability of finding a water molecule at a given distance r from the specified saccharide heteroatom, relative to the probability expected for a random distribution at the same distance. The random distribution of water molecules in the bulk solution will have a constant density, whereas the density of the water molecules close to the saccharide molecules will vary depending on the solute-solvent interactions. Hydrophobic regions of the solute molecule will induce a reduction in the solvent density whereas the contrary will be true for hydrophilic regions of the saccharide molecules. The heights of the peak in the RDF of **9mer-III** indicate that the residues E, I, D and H are less solvated than the residue A, probably due to favorable intramolecular interactions on a folded conformation, while RDF of **9mer-II** indicate than the residue H and I are equally solvated than the residue A, probably due to intramolecular interactions are practically absent on an unfolded conformation (Supplementary Fig. 2.3).

Also, RDF were calculated between selected oxygen atoms of the saccharides and all of the water molecules in order to characterize the overall solvation of **9mer-III** and **9mer-II**. The first sphere of nearest neighbors for both glycans is indicated by a maximum in g(r) at 2.4 Å and the limitations of the first sphere are indicated by a minimum in g(r) at 3.6 Å, and the second sphere is indicated by a maximum at 4.2 Å and the limitations of the second sphere are indicated by a minimum in g(r) at 5.6 Å (Supplementary Fig. 2.3).

To find the optimal choice for d, we calculated the scattering intensity of two conformations of both glycans using a specific envelope of 0.2, 0.3, 0.5, 0.7, 0.9 and 1.0 nm. In general, the scattering intensity calculated using different envelopes did not change significantly with increasing d, except in the case of the unfolded conformation of **9mer-III** where intensity at low-q converged with the increasingly bulk-like solvent behind the second solvation shell (Supplementary Fig. 3.2 and 3.3). Based on this study was constructed the envelope using d = 7 Å, in agreement with the value suggested by Park et al.[19]

Bibliography

(1) Fittolani, G.; Tyrikos-Ergas, T.; Poveda, A.; Yu, Y.; Yadav, N.; Seeberger, P. H.; Jiménez-Barbero, J.; Delbianco, M. Synthesis of a Glycan Hairpin. *Nat. Chem.* **2023**, *15* (10), 1461–1469.

(2) Yadav, N.; Djalali, S.; Poveda, A.; Ricardo, M. G.; Seeberger, P. H.; Jiménez-Barbero, J.; Delbianco, M. Dissecting the Conformational Stability of a Glycan Hairpin. *J. Am. Chem. Soc.* **2024**, *146* (9), 6369–6376.

(3) Van Der Spoel, D.; Lindahl, E.; Hess, B.; Groenhof, G.; Mark, A. E.; Berendsen, H. J. C. GROMACS: Fast, Flexible, and Free. *J. Comput. Chem.* **2005**, *26* (16), 1701–1718. https://doi.org/10.1002/jcc.20291.

(4) Kirschner, K. N.; Yongye, A. B.; Tschampel, S. M.; González-Outeiriño, J.; Daniels, C. R.; Foley, B. L.; Woods, R. J. GLYCAM06: A Generalizable Biomolecular Force Field. Carbohydrates. *J. Comput. Chem.* **2008**, *29* (4), 622–655. https://doi.org/10.1002/jcc.20820.

(5) Sauter, J.; Grafmüller, A. Predicting the Chemical Potential and Osmotic Pressure of Polysaccharide Solutions by Molecular Simulations. *J. Chem. Theory Comput.* **2016**, *12* (9), 4375–4384. https://doi.org/10.1021/acs.jctc.6b00295.

(6) Mahoney, M. W.; Jorgensen, W. L. A Five-Site Model for Liquid Water and the Reproduction of the Density Anomaly by Rigid, Nonpolarizable Potential Functions. *J. Chem. Phys.* **2000**, *112* (20), 8910–8922.

(7) Hoover, W. G. Canonical Dynamics: Equilibrium Phase-Space Distributions. *Phys. Rev. A* **1985**, *31* (3), 1695–1697. https://doi.org/10.1103/PhysRevA.31.1695.

(8) Nosé, S. A Unified Formulation of the Constant Temperature Molecular Dynamics Methods. *J. Chem. Phys.* **1984**, *81* (1), 511–519. https://doi.org/10.1063/1.447334.

(9) Parrinello, M.; Rahman, A. Polymorphic Transitions in Single Crystals: A New Molecular Dynamics Method. *J. Appl. Phys.* *52* (12), 7182–7190. https://doi.org/10.1063/1.328693.

(10) Parrinello, M.; Rahman, A. Crystal Structure and Pair Potentials: A Molecular-Dynamics Study. *Phys. Rev. Lett.* **1980**, *45* (14), 1196–1199. https://doi.org/10.1103/PhysRevLett.45.1196.

(11) Darden, T.; York, D.; Pedersen, L. Particle Mesh Ewald: An N·log(N) Method for Ewald Sums in Large Systems. *J. Chem. Phys.* **1993**, *98* (12), 10089–10092. https://doi.org/10.1063/1.464397.

(12) Hess, B.; Bekker, H.; Berendsen, H. J. C. 3 LINCS: A Linear Constraint Solver for Molecular Simulations. *J. Comput. Chem. 18* (12), 1463–1472. https://doi.org/10.1002/(SICI)1096-987X(199709)18:12%3C1463::AID-JCC4%3E3.0.CO;2-H.
(13) Miyamoto, S.; Kollman, P. A. Settle: An Analytical Version of the SHAKE and RATTLE Algorithm for Rigid Water Models. *J. Comput. Chem.* **1992**, *13* (8), 952–962. https://doi.org/10.1002/jcc.540130805.
(14) Chen, P.; Hub, J. S. Validating Solution Ensembles from Molecular Dynamics Simulation by Wide-Angle X-Ray Scattering Data. *Biophys. J.* **2014**, *107* (2), 435–447. https://doi.org/10.1016/j.bpj.2014.06.006.
(15) Hub, J. S. Interpreting Solution X-Ray Scattering Data Using Molecular Simulations. *Curr. Opin. Struct. Biol.* **2018**, *49*, 18–26. https://doi.org/10.1016/j.sbi.2017.11.002.
(16) Knight, C. J.; Hub, J. S. WAXSiS: A Web Server for the Calculation of SAXS/WAXS Curves Based on Explicit-Solvent Molecular Dynamics. *Nucleic Acids Res.* **2015**, *43* (W1), W225–W230. https://doi.org/10.1093/nar/gkv309.
(17) Chatzimagas, L.; Hub, J. S. Predicting Solution Scattering Patterns with Explicit-Solvent Molecular Simulations. *ArXiv Prepr. ArXiv220404961* **2022**.
(18) Doyle, P. T.; Turner, P. S. Relativistic Hartree-Fock X-Ray and Electron Scattering Factors. *Cryst. Phys. Diffr. Theor. Gen. Crystallogr.* **1968**, *24* (3), 390–397.
(19) Park, S.; Bardhan, J. P.; Roux, B.; Makowski, L. Simulated X-Ray Scattering of Protein Solutions Using Explicit-Solvent Models. *J. Chem. Phys.* **2009**, *130* (13), 134114. https://doi.org/10.1063/1.3099611.
